# Identification of potential transcription factors that enhance human iPSC generation

**DOI:** 10.1038/s41598-020-78932-9

**Published:** 2020-12-15

**Authors:** Nuha T. Swaidan, Salam Salloum-Asfar, Freshteh Palangi, Khaoula Errafii, Nada H. Soliman, Ahmed T. Aboughalia, Abdul Haseeb S. Wali, Sara A. Abdulla, Mohamed M. Emara

**Affiliations:** 1 Neurological Disorders Research Center, Qatar Biomedical Research Institute (QBRI), Hamad Bin Khalifa University (HBKU), Education City, Qatar Foundation (QF), Doha, Qatar; 2 Genomics Core Facility, Qatar Biomedical Research Institute (QBRI), Hamad Bin Khalifa University (HBKU), Education City, Qatar Foundation, Doha, Qatar; 3 College of Health and Life Sciences, Hamad Bin Khalifa University, Education City, Qatar Foundation, Doha, Qatar; 4 Basic Medical Sciences Department, College of Medicine, QU Health, Qatar University, Doha, Qatar; 5 Biomedical and Pharmaceutical Research Unit, QU Health, Qatar University, Doha, Qatar

**Keywords:** Molecular biology, Stem cells

## Abstract

Although many factors have been identified and used to enhance the iPSC reprogramming process, its efficiency remains quite low. In addition, reprogramming efficacy has been evidenced to be affected by disease mutations that are present in patient samples. In this study, using RNA-seq platform we have identified and validated the differential gene expression of five transcription factors (TFs) (*GBX2, NANOGP8, SP8, PEG3,* and *ZIC1*) that were associated with a remarkable increase in the number of iPSC colonies generated from a patient with Parkinson's disease. We have applied different bioinformatics tools (Gene ontology, protein–protein interaction, and signaling pathways analyses) to investigate the possible roles of these TFs in pluripotency and developmental process. Interestingly, *GBX2, NANOGP8, SP8, PEG3,* and *ZIC1* were found to play a role in maintaining pluripotency, regulating self-renewal stages, and interacting with other factors that are involved in pluripotency regulation including *OCT4, SOX2, NANOG,* and *KLF4*. Therefore, the TFs identified in this study could be used as additional transcription factors that enhance reprogramming efficiency to boost iPSC generation technology.

## Introduction

The concept of somatic cell reprogramming is based on the presence of a combination of factors and conditions to generate induced pluripotent stem cells (iPSCs)^1^. Several factors have been found to play a crucial role in reprogramming since the discovery of iPSCs of which the ectopic expression of *OCT3/4*, *SOX2*, *KLF4,* and *c-MYC* (OSKM) is known to be the most robust method^2–4^. Although the process of somatic cell reprogramming into a pluripotent state simply depends on ectopic expression of pluripotent genes, it remains inefficient and only a small number of cells can undergo complete reprogramming^5^.

The process of reprogramming and its efficiency depends on several factors which may influence a cell’s pluripotency, proliferation, or epigenetics. Previous investigations have identified numerous enhancers and barriers that regulate the reprogramming process^6^. Activation of such enhancers and inhibition of such barriers have been found to improve the reprogramming efficiency. *OCT3/4*, *SOX2* and *KLF4* are known to be the key regulators of pluripotency genes and inhibitors for genes that promote differentiation. Also, *NANOG* has been identified to be a core regulator of pluripotency and can be used along with *OCT3/4* and *SOX2* to induce reprogramming in somatic cells^5,7^. In addition to these core regulators, several transcription factors have been recognized to induce and enhance the reprogramming process including, (1) *NR5A2*^8^, (2) *UTF1*^9^, (3) *SALL4*^10^, (4) *FOXH1*^11^, and (5) *GLIS1*^12^. Furthermore, other factors related to cell proliferation and apoptosis such as *C-MYC*, cyclin D1 and suppressors of p53 were found to enhance reprogramming efficiency^13,14^.

A wide range of somatic cells can be used as a source for reprogramming and generating iPSCs^5,7^. However, cellular phenotypes and human mutations associated with different diseases, have been evidenced to influence the reprogramming efficiency, reprogramming factors required for iPSC induction and iPSC quality^15^. Indeed, these mutations could be vital to gain more understanding about the reprogramming mechanisms and hence help to improve reprogramming efficiencies. One of the diseases that is known to be correlated to gene mutations is Parkinson’s diseases (PD), which is generated as a result of the abnormal aggregation of alpha synuclein (α-syn) protein^16^. Several mutations have been reported in the gene encoding this protein and are highly associated with the familial form of the disease. α-syn point mutation (A53T) is considered the most frequent (~ 85%) and well-studied form, expressing high levels of pathogenicity and significantly increasing disease progression^17,18^. Beside these familial cases reported, sporadic ones that represent the majority of PD cases (90%)^19^ and occur randomly without a clear or definite cause, have remarkably displayed the pathological forms of α-syn^16^.

Altogether the previously mentioned data indicate the possibility that disease mutations could affect the transcription factor regulatory network(s); and thus, increase the likelihood of identifying novel factors that enhance the iPSC generation process. Indeed, in this study we aim to test this hypothesis in the context of PD using familial PD (A53T mutation) and sporadic PD patient samples. In this work, we were able to identify a number of transcription factors that may be involved in enhancing iPSC generation. We report a set of genes that were differentially expressed in generated iPSCs despite the presence or absence of disease associated mutation. Some of these changes were dramatic and coincides well with a robust increase in the generated iPSC colonies. Screening of the differentially expressed genes revealed *GBX2, NANOGP8, SP8, PEG3,* and *ZIC1* as possible transcription factors, which may enhance reprogramming. Furthermore, we provide evidence that those factors may be crucial in maintaining pluripotency and regulating self-renewal stages and thus could be used as pluripotency transcription factors for iPSC generation.

## Results

### Fibroblasts from PD patients carrying the same mutation showed different iPSC reprogramming capacity

**Figure 1:**
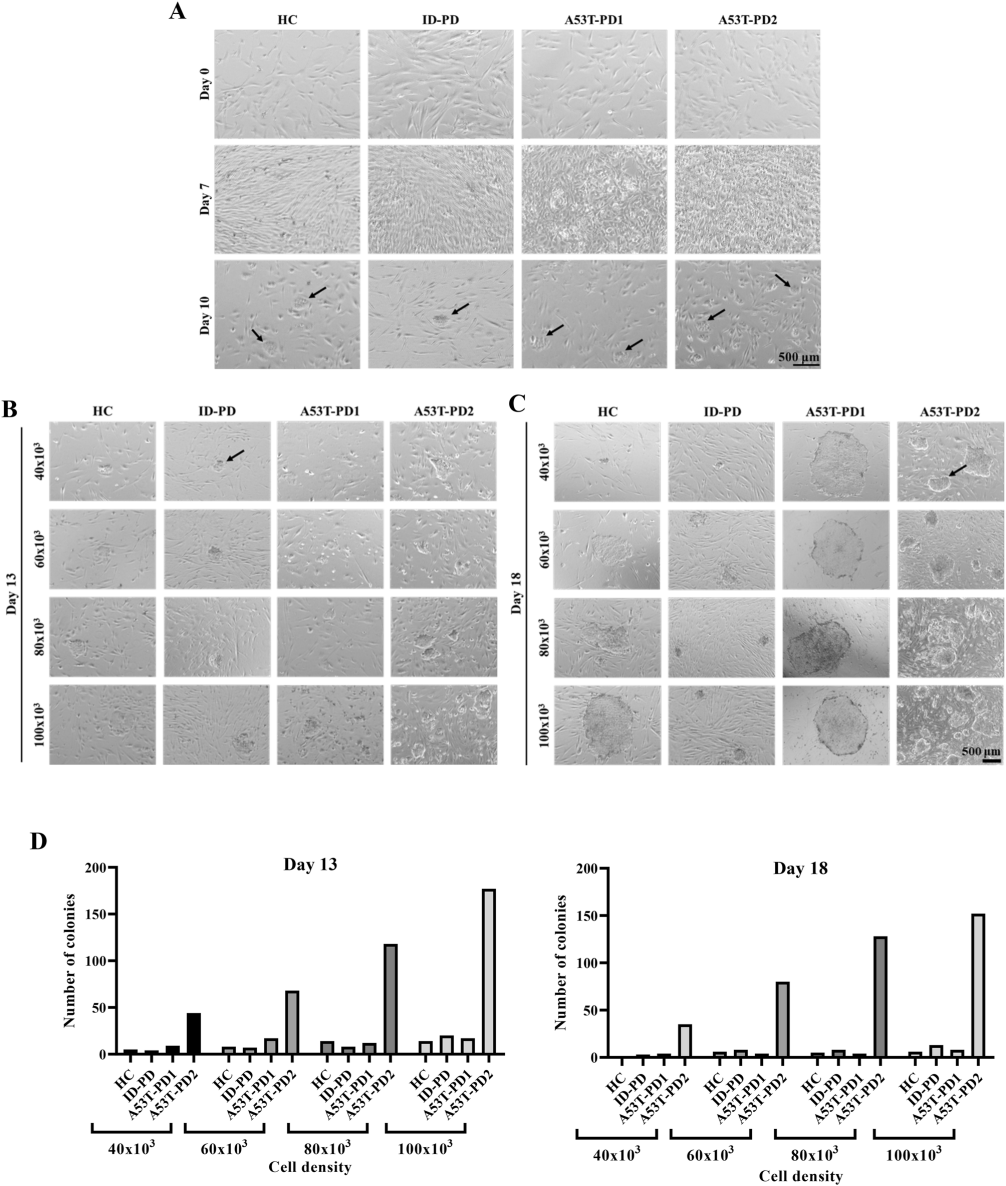
Fibroblasts from PD patients carrying the same mutation showed different iPSC reprogramming capacity. (**A**) Represents the early stages of control and PD fibroblast reprogramming. Fibroblast morphology was observed on day 0 and day 7 post transduction. After transferring the cells onto Matrigel coated plates, observable cell clumps have appeared on day 10 post transduction and are indicated by the black arrows. (**B**, **C**) Represents the reprogramming efficiency of the four reprogrammed samples. The shape and size of the emerged iPSC colonies in different cell densities were observed on day 13 (**B**) and day 18 (**C**) post transduction and the arrow in each figure indicates the minimum size of the counted colonies in each day. (**D**) Represents the number of emerged iPSC colonies plotted against the different cell densities for each reprogrammed sample on day 13 and 18; respectively.

As some mutations associated with human diseases have been found to affect reprogramming efficiency^15^, we tested the effect of A53T PD mutation on iPSC reprogramming. Reprogramming was done on dermal fibroblasts collected from four different human subjects. Two carrying the same mutation, A53T, designated as A53T-PD1 and A53T-PD2, one idiopathic with no mutation labeled as (ID-PD), to be used as PD non-mutant control, and one healthy control labeled as (HC). All fibroblast samples were transduced with reprogramming vectors and the morphological changes associated with iPSC generation were assessed. On day 10 post transduction, only A53T-PD2 sample showed a pronounced degree of cell clumping (Fig. 1A, lower panels, black arrows) indicating an early reprogramming event occurrence within this fibroblast.

To further test this effect, all transduced cells were re-plated at different cell densities and the total number and size of emerged iPSC colonies were determined at day 13 and 18 after transduction. As expected, with all cell densities, a gradual increase in the number of emerging colonies was observed in both days and was positively correlated with the increase in cell densities (Fig. 1B–D). However, A53T-PD2 showed a remarkable number of emerged colonies among the four transduced fibroblasts (Fig. 1B–D). Indeed, the reprogramming efficiency of A53T-PD2, assuming that we achieved 100% fibroblast transduction, reached to ~ 0.2%, whereas HC, ID-PD, and A53T-PD1 showed a reprogramming efficiency of not more than 0.02%. These results indicate variable reprogramming events that may be occurring in different fibroblasts regardless the presence or absence of the disease mutation.

### Generated iPSCs that showed different reprogramming capacity exhibited similar pluripotent characteristics

**Figure 2:**
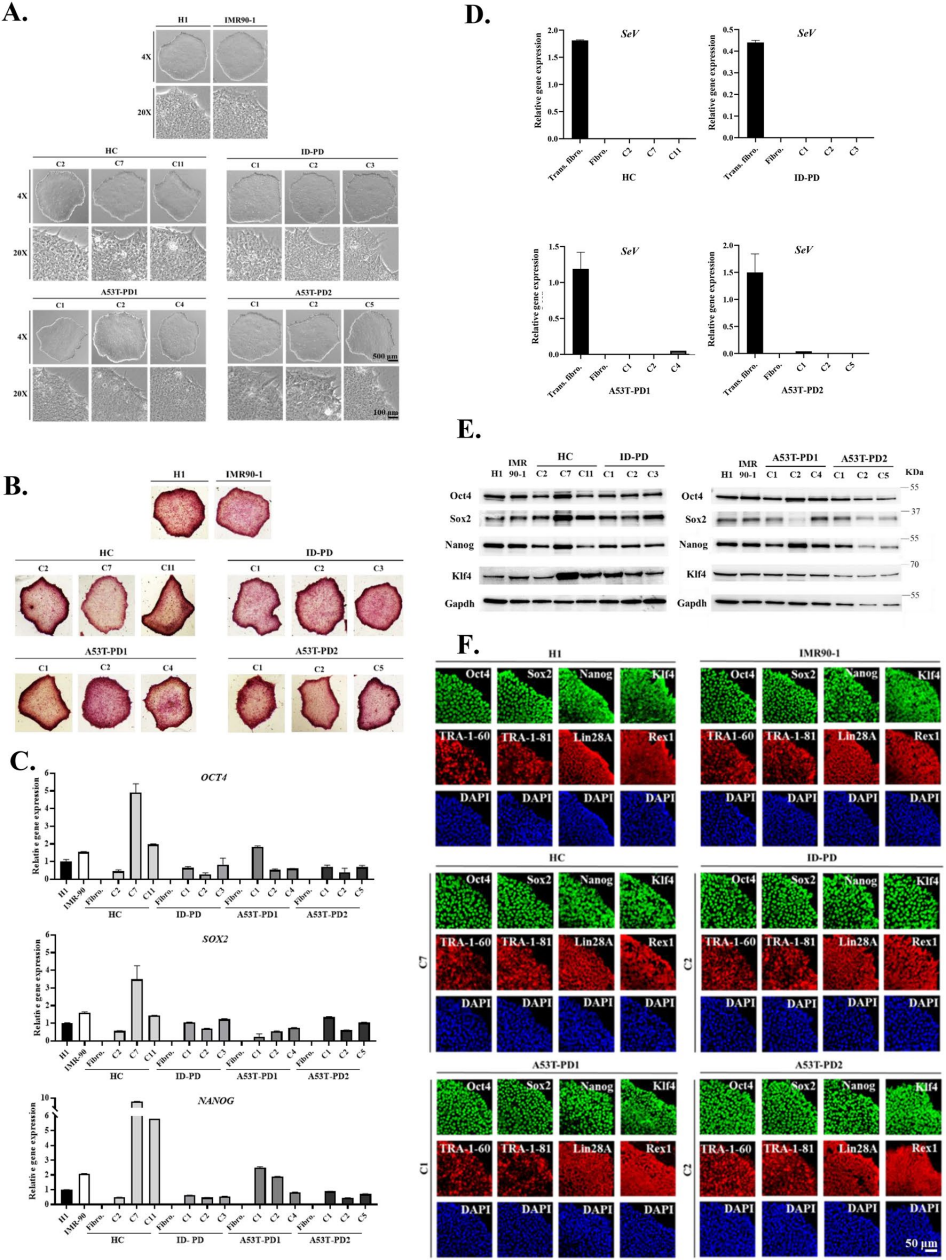
Generated iPSCs that showed different reprogramming capacity exhibited similar pluripotent characteristics. (**A**) Represents the ESC-like morphology of the established iPSCs in comparison to H1 and IMR90-1 cells which was observed by light microscopy using 2 different magnifications (× 4 and × 20). (**B**) Represents the alkaline phosphatase expression that was expressed by the established iPSC colonies compared to that expressed by H1 and IMR90-1 cells. Microscopic images were taken using × 10 magnification. (**C**) Representative graphs for RT-qPCR analysis of pluripotent genes that were expressed by the generated iPSCs and those expressed levels were compared with reference expressions represented by H1 and IMR90-1. (**D**) Representative graphs for expression levels of *SeV*-derived genes. Each graph represents one of the reprogrammed samples (HC, ID-PD, A53T-PD1 and A53T-PD2). Expression levels of pluripotent genes and *SeV*-derived genes were normalized by the expression of *GAPDH* as an endogenous control gene and the original fibroblasts were used as negative control for detected genes. Fibro: fibroblasts; Trans. fibro.: transduced fibroblasts on day 7 post transduction. (**E**) Represents Western blot analysis that shows the expression levels of pluripotent proteins [Oct4 (~ 45 KDa), Sox2 (~ 34 KDa), Nanog (~ 40 KDa), and Klf4 (~ 65 KDa)] that were expressed in the established iPSC colonies and in reference cells H1 and IMR90-1. Left panel shows the proteins expressed in HC and ID-PD colonies; while the right panel indicates the proteins expressed in A53T-PD1 and A53T-PD2 colonies. Gapdh was used as a loading control. Full-length blots are presented in Supplementary Fig. 2. (**F**) Representative immunofluorescence microscope images of pluripotent markers that were expressed by the generated iPSCs. [Oct4, Sox2, Nanog and Klf4] are represented in green, while [Lin28A, Rex1, TRA-1–60 and TRA-1–81] are represented in red. DAPI marks nuclei (blue). One representative colony from each reprogrammed sample in reference to H1 and IMR90-1 cells is illustrated in this figure. HC (C7), ID-PD (C2), A53T-PD1 (C1) and A53T-PD2 (C2).

To test whether the variations in reprogramming capacity will affect pluripotent characteristics of the generated iPSC clones, we performed a comparative characterization of those clones using morphological appearance, alkaline phosphatase (AP), immunocytochemistry, Western blotting, and RT-qPCR analyses (Fig. 2). Three iPSC clones from each reprogrammed sample were randomly chosen for characterization. Morphologically, all generated iPSC clones demonstrated similar prominent and homogenous edges colonies composed of densely packed compact cells with high nucleus to cytoplasm ratio, which is the typical stem cell appearance shown in the reference hESCs (H1) and hiPSCs (IMR90-1) colonies (Fig. 2A). This matching characteristics of the genuine iPSCs and the reference pluripotent stem cells was further observed in AP activity, regardless their reprograming capacity (Fig. 2B). In addition, the expression levels of the endogenous pluripotent genes (*OCT4*, *SOX2,* and *NANOG*) were comparable in all characterized clones as well as H1 and IMR90-1 cell lines (Fig. 2C). In contrast, fibroblast cells that were used as negative controls did not show any expression of those genes. This data indicates that all generated iPSC clones have the same morphological appearance, biochemical activities, and known pluripotent gene expression irrespective to their phenotype.

To exclude the possibility that the differences in iPSC cellular phenotypes that we observed in Fig. 1 are not due to the interference and the persistence of Sendai virus (*SeV)*-derived genes, we assessed the expression levels of those genes for each generated iPSCs, at passage 8, using RT-qPCR. As shown in Fig. 2D, all iPSC clones showed undetectable expression levels of *SeV* genes, which were similar to those observed in the non-transduced fibroblast samples. Although *SeV*-derived genes expression level of transduced ID-PD fibroblasts seemed to be lower (Fig. 2D), ID-PD and A53T-PD1 have the same rate of reprogramming, while A53T-PD2 has shown the highest rate of reprogramming. In addition to the gene expression levels assessment, we compared the expression of pluripotent proteins, Oct4, Sox2, Nanog and Klf4, in the generated clones as well as in H1 and IMR90-1 using Western blot analysis. Similar expression levels which are comparable to H1 and IMR90-1 were observed with generated iPSC clones (Fig. 2E). The same degree of pluripotency in the generated iPSC clones was also observed in immunocytochemistry analysis detecting the expression of pluripotent proteins (Oct4, Sox2, Nanog, Klf4 (Fig. 2F, green), TRA-1-60, TRA-1-81, Lin28A and Rex1 (Fig. 2F, red)). These data supported the pluripotency status of the characterized iPSC clones and confirmed that these clones generated from healthy individual and PD patients are iPSC per se. Taken together, these results suggest that there may be other genes that modulate the given variations in A53T-PD2 iPSC generation.

### RNA-sequencing identifies transcriptional changes between fibroblasts and iPSCs samples

**Figure 3:**
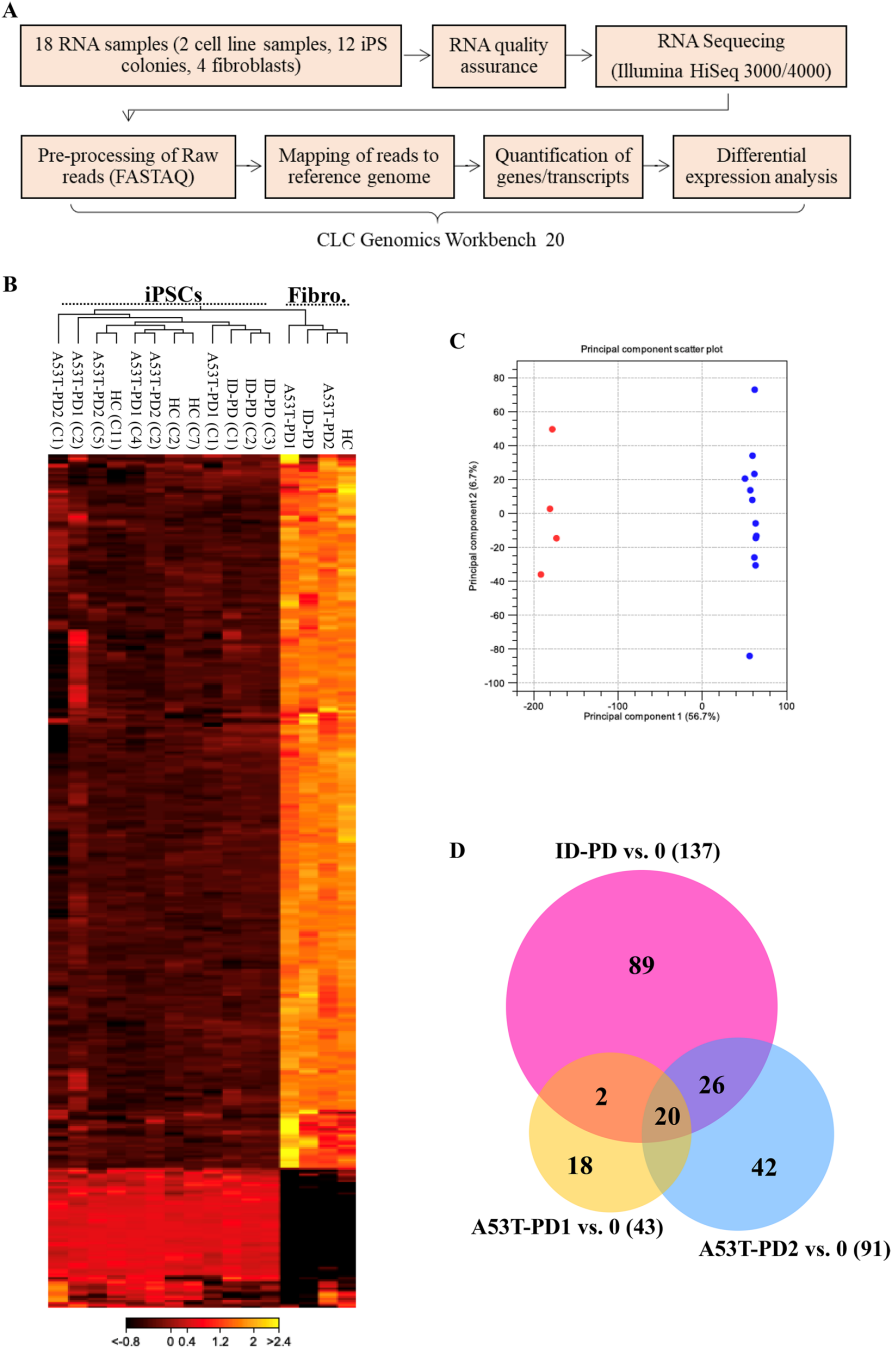
RNA-sequencing identifies transcriptional changes between fibroblasts and iPSCs samples. (**A**) Flowchart of the RNA-Seq and downstream analysis. (**B**) Heatmap analysis for the genes expression levels that showed unique cluster for iPS cell lines and another unique cluster for fibroblasts. (**C**) Two dimensional PCA analysis that shows similarities among fibroblasts and iPS cells; dots highlighted in red and blue represent fibroblasts and iPSCs gene sets; respectively. (**D**) Venn diagram of differentially expressed genes (FDR < 0.01, FC > 2) in the PD iPS cell lines (ID-PD, A53T-PD1 and A53T-PD2) versus control iPS cell line. “0” refers to IMR90-1.

RNA sequencing platform was used to characterize the mRNA of both fibroblasts and iPS cell lines (N = 3 for each sample) and to further identify possible genes other than the main pluripotent regulatory genes responsible for the variations in A53T-PD2 iPSC generation (Fig. 3A). Before sequencing, RNA quality and integrity was assessed using the Agilent Bioanalyzer 2100. All samples showed a clear peaks at 18S and 28S rRNA with RNA integrity number (RIN) of more than 8, which indicates intact RNA (Supplementary Fig. 1). RNA samples were sequenced using Illumina HiSeq 3000/4000. FastQC reports showed good quality of high throughput sequence data. Fastq files generated were later assembled and analyzed using the QIAGEN CLC Genomics Workbench v20 software. Around 90% of raw reads were uniquely mapped to the reference in all samples (Supplementary Table S1). Heatmap analysis of gene expression levels among iPS cell lines (N = 3 for each) and fibroblasts (Fig. 3B) showed a unique and distinguishable cluster of iPS cell lines from the unique cluster of fibroblasts. Another way to represent how genes in fibroblasts and iPS cell lines are expressed is the two-dimensional PCA analysis (Fig. 3C). The highlighted dots with red and blue represent fibroblasts and iPSCs gene sets respectively, and they illustrate similarities among fibroblasts and iPS cell lines as well as two unique clusters, which are fibroblast cluster and iPS cluster. Finally, Venn diagram has shown differentially expressed genes in the PD iPS cell lines (ID-PD, A53T-PD1 and A53T-PD2) (N = 3 each) versus control iPSCs (IMR90-1) (FDR < 0.01, Fold Change > 2). The genes that correspond to the Venn diagram are listed in (Supplementary Table S3) and (Fig. 3D) represents the Venn diagram that showed only 20 common genes between the three PD iPS cell lines, 2 common genes between A53T-PD1 and ID-PD iPS cell lines, 26 common genes between A53T-PD2 and ID-PD iPS cell lines and 3 common genes between A53T-PD1 and A53T-PD2 iPS cell lines. While 89, 18, and 42 genes were differentially expressed in ID-PD, A53T-PD1 and A53T-PD2, respectively.

### A set of genes involved in transcription regulation and development-related process are differentially expressed in A53T-PD2 iPS cell lines

**Figure 4:**
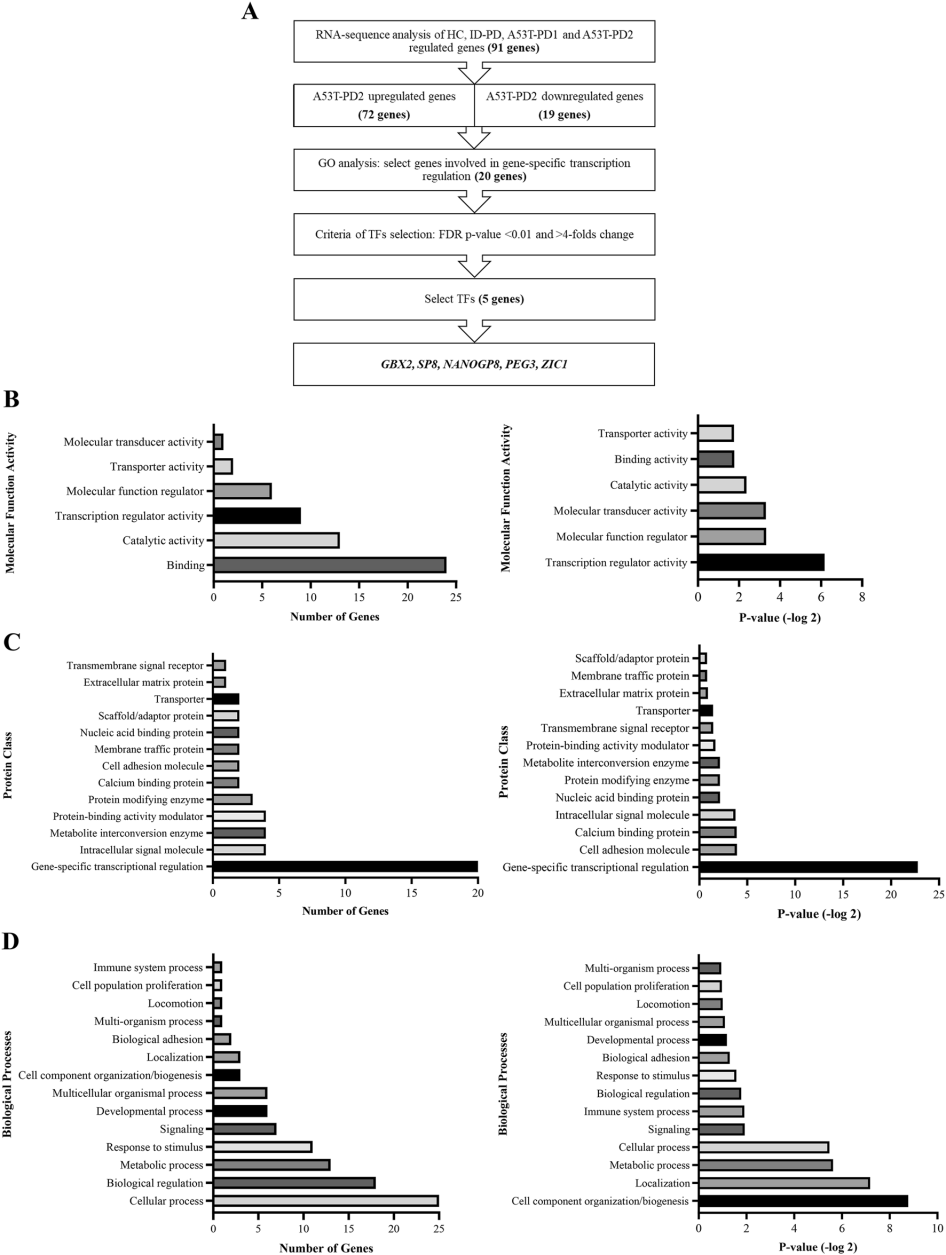
A set of genes involved in transcription regulation and development-related process are differentially expressed in A53T-PD2 iPS cell lines. (**A**) Flowchart for the process that led to the identification of five differentially expressed genes in A53T-PD2 iPS cell line. (**B**, **D**) Representative graphs for the GO analysis that classified the 91 differentially expressed genes according to their role and function within the cell (**B**, **D** left graphs). Right graphs (**B**, **D**) represent the statistical significance of those classified genes. (**C**) Representative graph that shows the protein classification for a specific number of genes (left graph). Right graph represents the statistical significance of those genes.

Given that A53T-PD2 showed the highest reprogramming capacity (Fig. 1), we were interested to identify significant differentially expressed candidate genes within this iPS cell lines (N = 3). Using the RNA sequencing data (Fig. 3 and Supplementary Table S2), differential gene expression levels of A53T-PD2 were compared to those expressed in the other generated iPS cell lines, HC, ID-PD, and A53T-PD1 (N = 3 each). We selected only the genes that showed an FDR ≤ 0.05 in A53T-PD2 and we identified 91 genes of which 72 genes were upregulated, while 19 genes were downregulated (Fig. 4A and Supplementary Table S3). Then, we applied Gene Ontology analysis (GO; http://www.pantherdb.org) to identify and classify the 91 differentially expressed genes according to their role and function within the cell (Fig. 4A). This analysis was based on selecting GO annotation results that link the regulated genes to the most common molecular and cellular functions related to pluripotency (transcription regulation and development-related process). Although, developmental-process related genes did not reach statistical significance (*p* value = 0.44) (Fig. 4D), gene-specific transcription regulators in both molecular function and protein classification were statistically significant (*p* value < 0.001). Therefore, we based our selection on the classification of molecular function and protein class, focusing on gene-specific transcription regulators (Fig. 4B,C). Indeed, we were able to identify 20 genes that fit our criteria which were classified into two groups. The first group has included eleven zinc finger transcription regulator genes, while the remaining nine genes (*SP8, FOXC1, NANOGP8, LMX1B, ZIC1, MLLT6, VENTX, GBX2,* and *PEG3)* were involved in other different transcription regulation activities.

**Figure 5:**
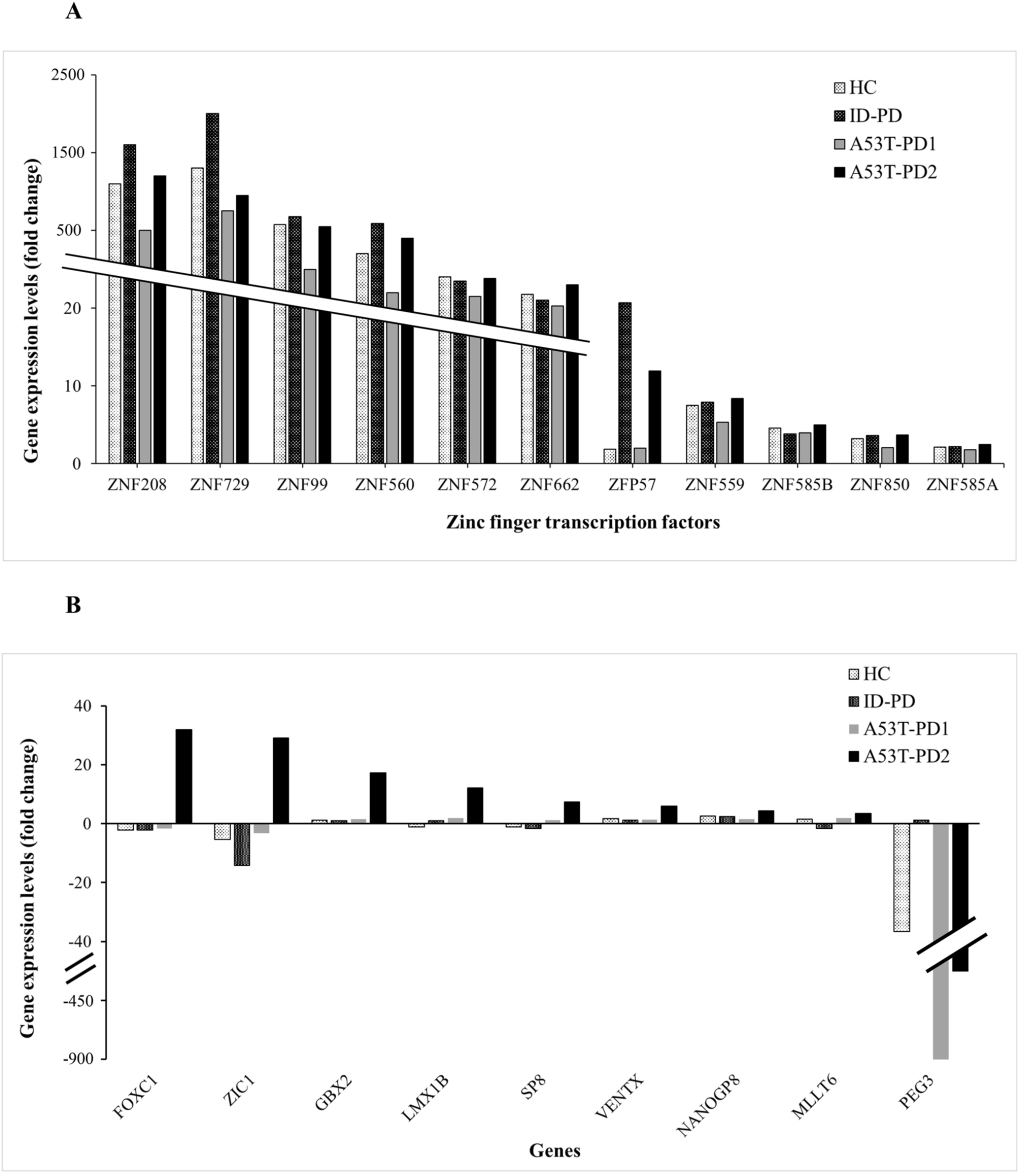
A set of transcription regulator genes may play a role in iPSC pluripotency regulation. Gene expression levels for the selected 20 transcription regulator genes were analyzed based on RNA-Seq results and represented into two graphs. (**A**) Represents the expression levels of the 11 zinc finger transcription factors expressed by the established iPS cell lines. (**B**) Represents the expression levels of the other 9 transcription regulator genes in the generated iPS cell lines.

Further deep analysis was applied to the expression levels of each of the twenty genes in each iPS cell line using the generated differentially expression data compared to the control iPSCs (IMR90-1) (Fig. 5). All zinc finger genes were upregulated in all cell lines with statistically significant FDR < 0.01 (Fig. 5A). For the rest of genes listed in the other transcription regulators group (Table 1), the highest upregulation was observed with *FOXC1* and *ZIC1* genes in A53T-PD2 iPS cell lines (N = 3) as compared to the other generated iPSCs . *FOXC1* increased 32 folds (significant FDR = 0.013), whereas *ZIC1* was upregulated 29 folds (significant FDR = 0.0075) (Fig. 5B; Supplementary Table S3). In contrast, both genes were downregulated in HC, ID-PD, and A53T-PD1 iPSCs (Fig. 5B; Supplementary Table S3). In addition, *GBX2, LMX1B*, *SP8, VENTX*, and *NANOGP8* genes were significantly upregulated only in A53T-PD2 iPS cell lines (17.15 folds, FDR < 0.0001; 12.11 folds, FDR = 0.034; 7.37 folds, FDR = 0.0014; 5.89 folds, FDR = 0.016; and 4.42-folds, FDR = 0.0004, respectively), but did not show any significant changes in other cell lines (Fig. 5B; Supplementary Table S3). On the other hand, *MLLT6*, which exhibited a similar trend of upregulation in A53T-PD2 (3.43-folds; FDR = 0.015), was slightly upregulated in HC (1.48 folds) and A53T-PD1 (1.91 folds), but downregulated in ID-PD (-1.6 folds). However, these fold changes did not reach any statistical significance (Fig. 5B; Supplementary Table S3). In contrast to all genes mentioned above, *PEG3* was significantly downregulated in all generated iPS cell lines except ID-PD. Remarkably such downregulation was very significant in A53T-PD2 (428.117 folds; FDR > 0.0001) and A53T-PD1 (898.08 folds; FDR = 3.3E-10) when compared to the downregulation occurred in HC iPSCs (36.61 folds; FDR > 0.052). Those results suggest that some of these genes might play a role in regulating pluripotency in iPSCs and hence reprogramming efficiency.

**Table 1:** Nine genes involved in transcription regulation (genes information are based on PANTHER classification system).

Gene ID	Gene name/gene symbol/ortholog	PANTHER family/PANTHER subfamily	PANTHER protein class	Species
HGNC:23112	Protocadherin fat 3/*FAT3*/ortholog	FAT ATYPICAL CADHERIN-RELATED/PROTOCADHERIN FAT 3	Cadherin	Homo sapiens
HGNC:3800	Forkhead box protein C1/*FOXC1*/ortholog	FORKHEAD BOX PROTEIN/FORKHEAD BOX PROTEIN C1	Winged helix/forkhead transcription factor	Homo sapiens
HGNC*:*4186	Homeobox protein GBX-2/*GBX2*/ortholog	HOMEOBOX PROTEIN GBX/HOMEOBOX PROTEIN GBX-2	Homeodomain transcription factor	Homo sapiens
HGNC:6654	LIM homeobox transcription factor 1-beta/*LMX1B*/ortholog	LIM/HOMEOBOX PROTEIN LHX/LIM HOMEOBOX TRANSCRIPTION FACTOR 1-BETA	Homeodomain transcription factor	Homo sapiens
HGNC:7138	Protein AF-17/*MLLT6*/ortholog	PHD FINGER PROTEINS/PROTEIN AF-17	Zinc finger transcription factor	Homo sapiens
HGNC:8826	Paternally-expressed gene 3 protein/*PEG3*/ortholog	ZINC FINGER PROTEIN/PATERNALLY-EXPRESSED GENE 3 PROTEIN	C2H2 zinc finger transcription factor	Homo sapiens
HGNC:12872	Zinc finger protein ZIC 1/*ZIC1*/ortholog	ZINC FINGER PROTEIN ZIC AND GLI/ZINC FINGER PROTEIN ZIC 1	C2H2 zinc finger transcription factor	Homo sapiens
HGNC:13639	Homeobox protein VENTX/*VENTX*/ortholog	HOMEOBOX PROTEIN/HOMEOBOX PROTEIN VENTX	Homeodomain transcription factor	Homo sapiens
HGNC:19196	Transcription factor Sp8/*SP8*/ortholog	KRUEPPEL-LIKE TRANSCRIPTION FACTOR/TRANSCRIPTION FACTOR SP8	C2H2 zinc finger transcription factor	Homo sapiens

### Gbx2, Nanogp8, Sp8, Peg3, and Zic1 are possibly playing a role in iPSC reprogramming

**Figure 6:**
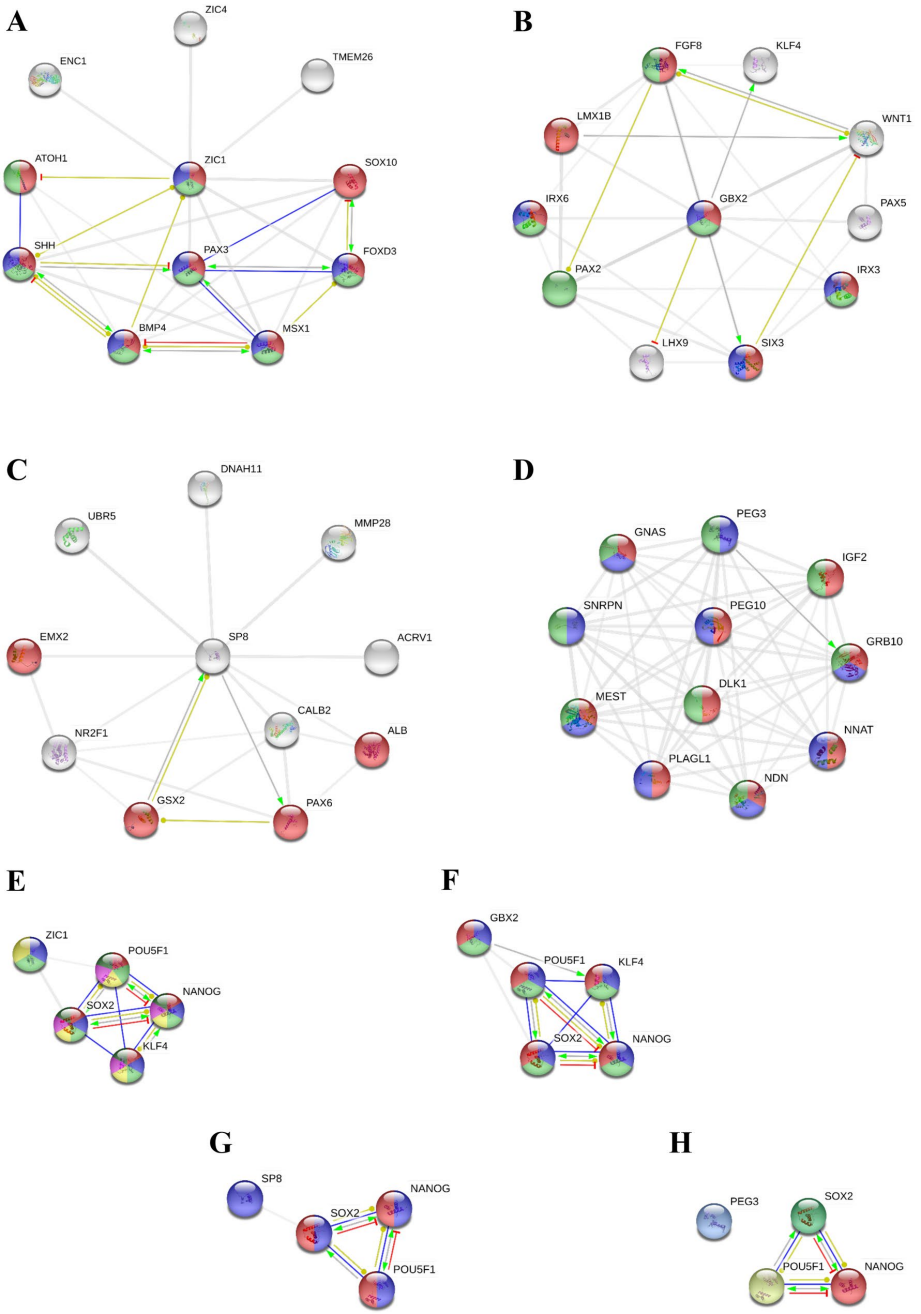
Gbx2, Nanogp8, Sp8, Peg3, and Zic1 are possibly playing a role in iPSC reprogramming. (**A**–**D**) Representative figures for protein–protein interaction networks as analyzed by String software. Each figure represents the possible proteins that could interact with each of the selected proteins Zic1 (**A**), Gbx2 (**B**), Sp8 (**C**), and Peg3 (**D**). (**E**–**H**) Each figure represents the predicted interaction network of each of the selected proteins Zic1 (**E**), Gbx2 (**F**), Sp8 (**G**), and Peg3 (**H**) with the globally known pluripotent regulatory proteins (Sox2, Pou5f1 (Oct4), Nanog and Klf4). No protein–protein interactions for Nanogp8 were found. Each network node represents all the proteins produced by a single, protein-coding gene locus. The colored nodes represent query proteins and first shell of interactors, while the white nodes indicate the second shell of interactors. Also, empty nodes mean that proteins are of unknown 3D structure, while the filled nodes indicate that some 3D structure is known or predicted. Moreover, protein associations are meant to be specific and meaningful; however, they do not necessarily mean that proteins are physically binding to each other. The network edges that are present in this figure are indicating the molecular action between the interacted proteins, where line shape indicates the predicted mode of action. Each line color represents a type of action including binding (blue line), inhibition (red line) and transcriptional regulation (yellow line). While the unidentified interactions are presented as grey lines. Finally, arrowed line means positive effect, bar-headed line means negative effect and circular-ended line indicates unspecified effect.

To make a final selection of the candidate genes that may be involved in producing the high number of A53T-PD2 iPS colonies, we set two stringent selection criteria from our initial analysis (Supplementary Table S3); an FDR < 0.01 and a fold change should be greater than 4 folds. Based on these criteria, five genes *GBX2, SP8, ZIC1, PEG3,* and *NANOGP8,* were selected and subjected to further analysis and validation (Table 2). We then used STRING software to predict proteins that could possibly interact with each of the selected proteins. *ZIC1* that was significantly upregulated (29 folds increase in A53T-PD2), was predicted to inhibit the expression of *ATOH1* gene, which plays a role in the differentiation of subsets of neural cells by activating E box-dependent transcription (Fig. 6A). Moreover, a mutual transcriptional regulation was observed between Zic1 and sonic hedgehog (Shh) protein, which has a role in cell growth and specialization. In addition, Bmp4 which is involved in self renewal of embryonic stem cells, was observed as a transcriptional regulator of *ZIC1*. Notably, Zic1 is predicted to have a number of unidentified interactions with different proteins; which are involved in pluripotency regulation (Foxd3*)* and other type of proteins (Zic4*,* Enc1*,* Tmem26*,* Sox10*,* Pax3 and Msx1) (Fig. 6A).

**Table 2:** Five selected genes based on fold change and FDR.

Gene	Fold changes (ID-PD vs control)	Fold changes (A53T-PD1 vs control)	Fold changes (A53T-PD2 vs control)
*GBX2*	1.00	1.46	17.16 (FDR < 0.001)
*ZIC1*	− 14.23	− 3.17	29.01 (FDR 0.008)
*SP8*	− 1.68	1.16	7.38 (FDR 0.001)
*PEG3*	1.12	− 898.08	− 428.12 (FDR < 0.001)
*NANOGP8*	2.36	1.49	4.42 (FDR < 0.001)

Since Foxd3 protein, has been found to be directly involved in the expression of Oct4*,* Sox2 and Nanog*,* it was of our interest to analyze the possibility of Zic1 interactions with pluripotent proteins. We used the globally known transcriptional factors proteins in maintaining pluripotency (Sox2, Oct4, Nanog, and Klf4) in our analysis. Zic1 is predicted to have two unidentified interactions with Sox2 and Oct4 but not with Nanog or Klf4 (Fig. 6E). We then applied GO within the same software and we confirmed that *ZIC1* is involved in transcription regulation. The protein possesses the ability to positively regulate the transcription that is DNA-dependent (represented in Fig. 6A with the red colored node) and by RNA polymerase II (represented in Fig. 6A with the green colored node), both activities have an FDR < 0.01. Interestingly, Zic1 interactors have been found to be involved in both transcription regulation activities except Sox10 that exhibited only DNA-dependent transcription activity (Fig. 6A).

Similarly, *GBX2*, which showed a 17 folds increase, has shown to be inhibiting the expression of *LHX9*, which is a LIM/homeobox protein and plays a role in gonadal development (Fig. 6B). In addition, Gbx2 was shown to activate *KLF4* gene, which plays an important role in maintaining embryonic stem cells, and in preventing their differentiation (Fig. 6B). Next, we analyzed the possibility of Gbx2 to interact with the globally known transcriptional factors in maintaining pluripotency. Beside the confirmed Gbx2 and Klf4 interaction that we observed in Fig. 6B, Gbx2 was found to have unidentified interactions with Oct4 and Sox2 (Fig. 6F). In contrast, no possible interaction was predicted with Nanog (Fig. 6F). To further confirm these results, GO analysis showed that *GBX2* is notably involved in pluripotency. We predicted Gbx2 along with the Sox2, Oct4, Nanog and Klf4, as a possible promoter of reprogramming and retention of pluripotency (represented in Fig. 6F with red colored node), a transcription activator by binding to a specific sequence of DNA that is part of the transcription regulatory region (represented in Fig. 6F with green colored node), and a positive transcription regulator by RNA polymerase II (represented in Fig. 6F with blue colored node). All these predictions were highly significant recording an FDR of 9.26e−12, 3.34e−06, 5.86e−06; respectively. In contrast, Sp8 did not show any possible interaction with proteins that are involved in pluripotency except with Sox2 (Fig. 6G). In addition, the Sp8 protein is significantly involved in maintaining totipotent embryonically restricted ground state (represented in Fig. 6C with red colored node) which has an FDR of 3.18e−05.

Interestingly, *PEG3* which was significantly downregulated in the gene expression analysis (Table 2) is predicted to activate Grb10 protein, which is involved in developmental processes (represented in Fig. 6D with red colored node of Grb10 protein, with an FDR of 0.0421). In addition, Peg3 had many unknown interactions with other developmental proteins including Peg10, Igf2, Gnas, Mest, Plagl1, Nnat, Ndn and Dlk1 (Fig. 6D). In contrast to the previous three proteins, Peg3 did not show any possible interaction with the globally known reprogramming transcription factors (Fig. 6H). Finally, we could not find any results for the protein–protein interactions for Nanogp8 in homo-sapiens (humans). These results indicate the possible interactions of Gbx2, Sp8, Zic1, and Peg3with pluripotent regulatory proteins and hence a possible role in reprogramming.

### GBX2, SP8, PEG3, and ZIC1 are differentially expressed in A53T-PD2 iPS cell line

**Figure 7:**
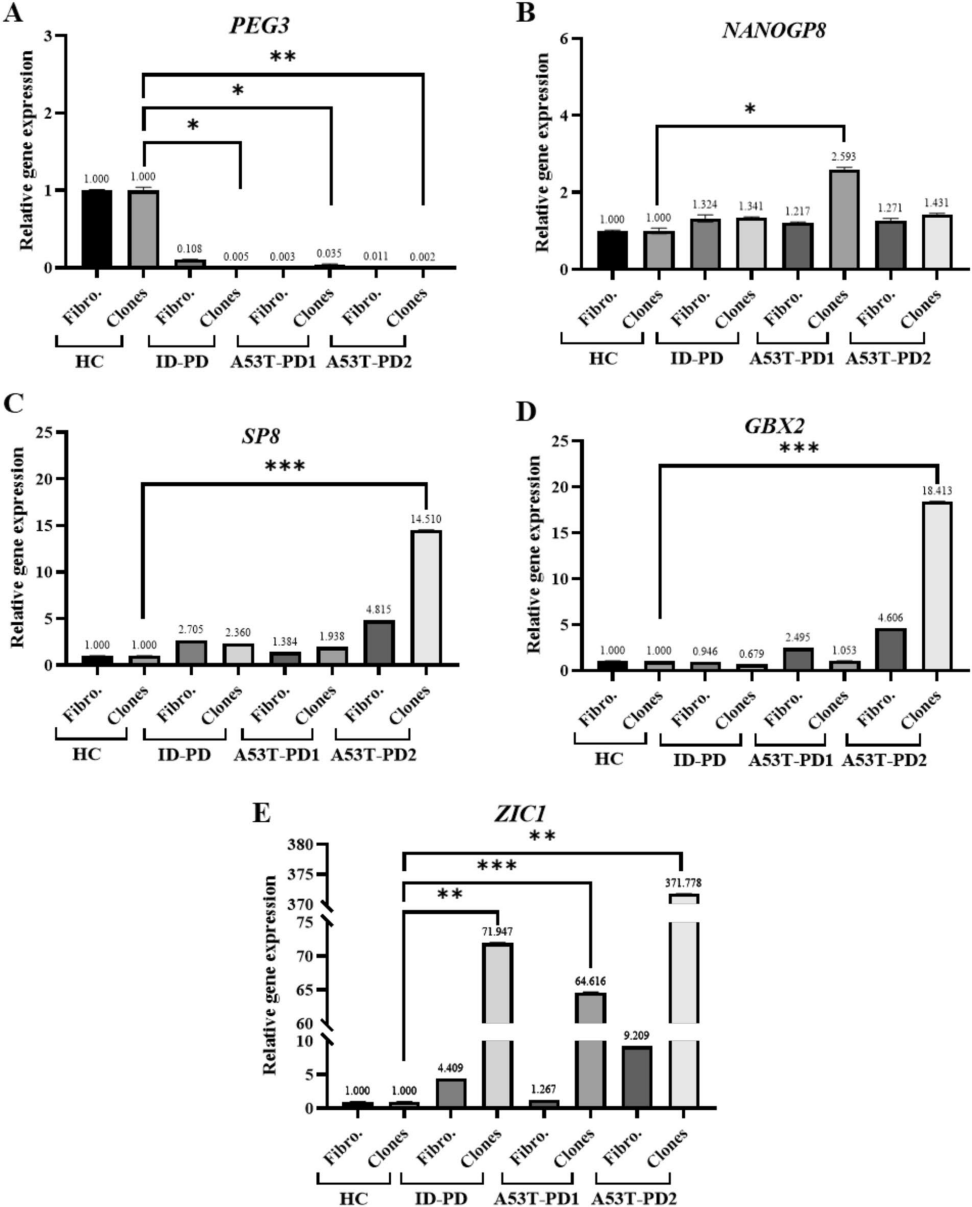
RT-qPCR analysis for the five selected genes supported the possible role of those genes in iPSC reprogramming. (**A**–**E**) Representative graphs for RT-qPCR analysis of the five nominated genes (*PEG3, NANOGP8, SP8, GBX2 and ZIC1*) that were expressed by both fibroblasts and generated iPS cell lines. Relative gene expression was normalized to *GAPDH* expression. Fibro: fibroblasts. Statistical significance was tested using the Mann–Whitney U test; ns: non-significant; *P < 0.05; **P < 0.01; ***P < 0.001. Results represent triplicates with similar results.

As a validation to RNA-seq analysis that nominate five genes for a possible role in iPSC reprogramming (*PEG3, NANOGP8, SP8, GBX2 and ZIC1*), we quantify their expression levels in both fibroblast and iPS cell lines using RT-qPCR; where the expression of these genes was relatively compared to control samples. A noteworthy point is that RNA-seq and RT-qPCR analysis for gene expression levels may show some insignificant differences. Among those tested genes, *PEG3* gene had the lowest significant expression levels in A53T-PD2 clones (N = 3) (500X lower) (Fig. 7A). In contrast, A53T-PD2 clones have shown significant higher expression levels of *SP8* (14X)*, GBX2* (18X) and *ZIC1* (370X) genes in comparison to the other generated iPS cell lines ID-PD (2X), (1.5X lower), (72X) and A53T-PD1 (2X), (1X), (65X), respectively. Both ID-PD and A53T-PD1 iPS cell lines have exhibited a downregulation and an upregulation in *GBX2* and *ZIC1;* respectively (Fig. 7C–E). While *SP8* was upregulated in A53T-PD1 and downregulated in ID-PD (Fig. 7C). Lastly, *NANOGP8* expression was significantly upregulated only in A53T-PD1 iPS cell lines (N = 3) (2.5X) (Fig. 7B). These results correlated well with our RNA-seq analysis and support our observation that suggests a potential role of additional transcription factors in increasing the number of iPS colonies in A53T-PD2 sample.

## Discussion

One can argue that a major limitation and setback of utilizing iPSC technology is its low reprogramming efficiency (0.1–1%)^7^. Therefore, it is crucial to identify robust reprogramming factors that increase the efficiency of generating iPSCs, improve the quality of reprogramming, and hasten the reprogramming time. It has been postulated that mutations associated with human diseases may affect the efficiency of reprogramming^15^, indicating that these mutations potentially influence the transcription factor networks that regulate reprogramming. Our data suggests that this is not necessarily the case, as we were able to generate a robust number of iPSC colonies from one PD patient carrying an A53T mutation (A53T-PD2), but not from the other patient carrying the same mutation (A53T-PD1). Furthermore, a previous study has shown that donor age negatively affects reprogramming efficiency and iPSC generation^20^, indicating that more youthful individuals harbor transcription factors that may enhance the reprogramming efficiency. Since PD is a neurodegenerative disease that has a tendency to appear in individuals younger than 50 years of age (early onset) as well as those that are older than 50 (late onset), we hypothesized that age difference could possibly be the reason behind the significant increase in the number of generated colonies observed in A53T-PD2. However, this possibility was excluded, because both A53T-PD2 and A53T-PD1 were isolated from early onset PD patients of 43 and 48 years old, respectively.

Our data suggests that neither a specific disease mutation nor patient’s age, has substantial effect on the capacity of iPSC generation. This consequently raises the possibility that transcription factor(s) (TF), specific to A53T-PD2, are highly regulated and thus increases the number of colonies observed and the ability to maintain a high degree of pluripotency. Indeed, our hypothesis was supported by the transcriptomic analysis data, which identified five transcriptional factors that were differentially expressed in A53T-PD2 and play a role in pluripotency as well as developmental process *(GBX2, ZIC1, NANOGP8, SP8* and *PEG3*) (Figs. 5B and 7). Among those genes, *GBX2* was highly upregulated in A53T-PD2 iPS cell lines (17 folds; FDR < 0.01) (Table 2 and Supplementary Table S2) as compared to other iPS cell lines generated under the same conditions. In fact, *GBX2* pluripotency-related function has been confirmed by several previous studies performed on mouse models. Tai et al. reported *Gbx2* to be a downstream TF involved in the LIF/Stat3 signaling pathway, which is known to be sufficient in maintaining pluripotency of mouse embryonic stem cells (mESCs)^21^. *Gbx2* is not only involved in pluripotency maintenance, but also is capable of reverting differentiated cells back to its naïve pluripotent state, indicating its importance in promoting reprogramming^21^.

The capability of *GBX2* to maintain pluripotency and direct cells towards stemness is explained by its role in activating one of the well-known pluripotent proteins, Klf4, which is also part of the LIF/Stat3 pathway^22^. This postulated mechanism is consistent with our analysis, which identified *GBX2* as a transcription factor (Fig. 4) that regulates pluripotency and acts as a direct activator of *KLF4* expression (Fig. 6B,F). Although, we did not carry out functional studies to confirm the role of *GBX2* in reprogramming and maintaining pluripotency, it has been previously shown that *Gbx2* overexpression inhibits down-regulation of *Klf4* in mESCs, whereas gene knock-down decreases *Klf4* expression levels^22^. Furthermore, *Gbx2* and *Klf4* knock-down in mouse epithelial stem cells (mEpiSCs) leads to either death or differentiation of mEpiSCs^22^. Our data, along with these results, confirm our prediction that *GBX2* plays a role in enhancing somatic cell reprogramming and maintaining self-renewal, possibly through a synergistic effect with *KLF4* or direct upregulation of *KLF4*. It is important to note that, *GBX2* has also been evidenced to be expressed in other species like rabbits. *Gbx2* is strongly expressed in rabbit ESCs and is considered to be a naïve pluripotent gene and a marker of pluripotency^23^. It is highly possible that the scenario that takes place in mouse and rabbit ESCs will also occur in hESCs and hiPSCs. Additionally, *GBX2* was reported as a naïve pluripotent marker along with *KLF4, NANOG* and *MYC*, which drive the transition of EpiSCs to ESCs^24–26^. Altogether, this suggests that Gbx2 is an essential pluripotent protein that promotes cell reprogramming and maintains self-renewal.

**Figure 8:**
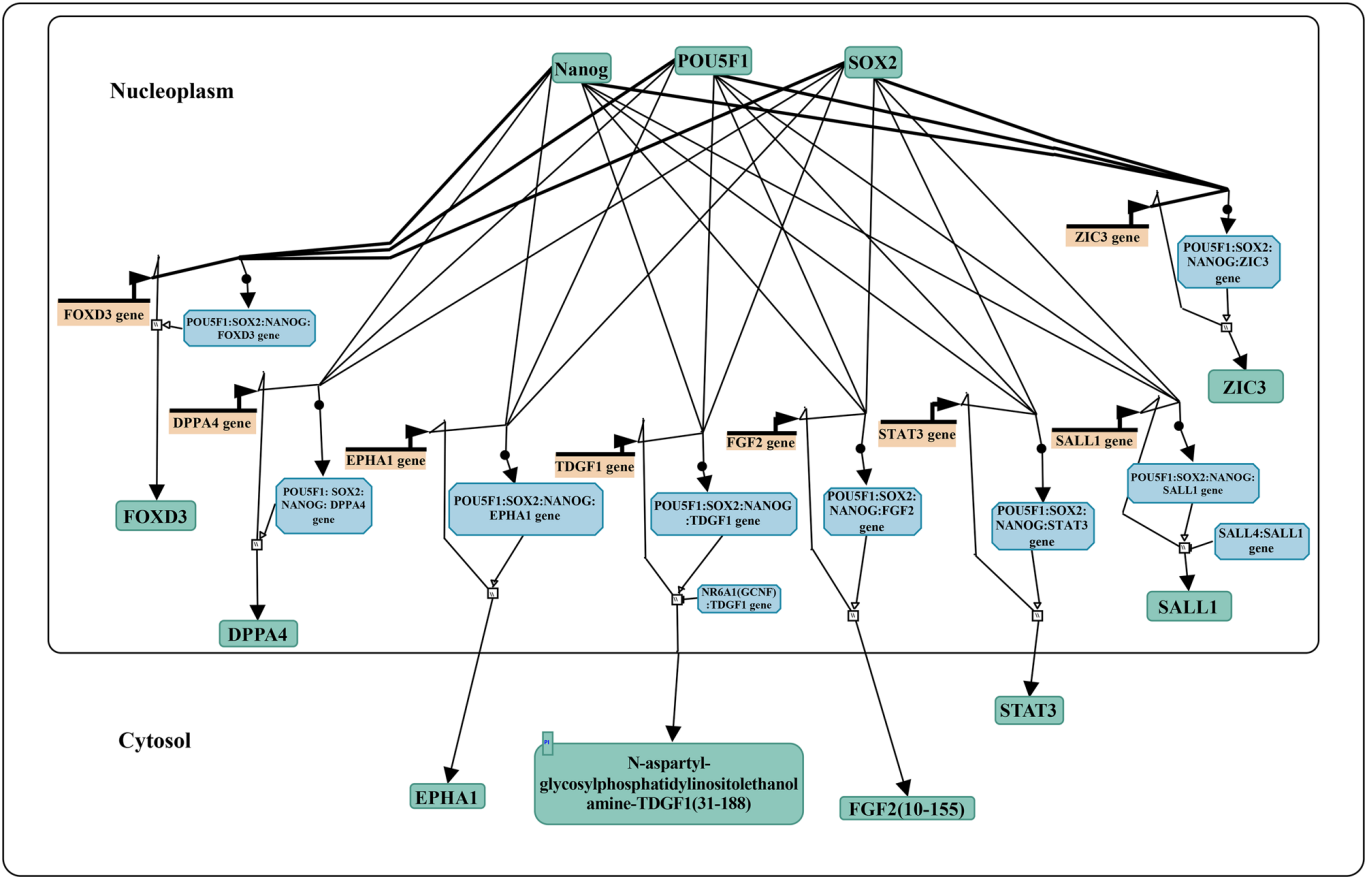
Both *FOXD3* and *ZIC3* are involved in pluripotency regulation pathway. The figure has been obtained and modified under CC-BY 4.0 international license from Reactome. An interactive version with links to detailed pathway representation is accessible https://reactome.org/content/detail/R-HSA-2892247. It shows that NANOG, POU5F1 (OCT4) and SOX2 bind to the promoters of different target genes which further activates or suppresses the expression of those target genes^43–46^. The mentioned genes in this figure have been described to be involved in the core transcriptional network of pluripotent stem cells^47–49^.

*ZIC1* showed the highest upregulation, 29 folds (FDR < 0.01), in A53T-PD2 iPS cell lines compared to other iPS cell lines (Table 2 and Supplementary Table S2). In line with our results, it has been previously shown that *ZIC1* is remarkably upregulated along with *SOX2*, *OCT4* and *KLF4*, supporting the prediction that *ZIC1* might activate the well-known genes related to self-renewal pathways^27^. In addition, transcriptome analysis of molecular pluripotency markers showed ZIC1 expression in primed pluripotent ESCs derived from bovine blastocysts^28^. Through STRING, we were able to predict an interaction between Zic1 and Foxd3, which plays a role in pluripotency signaling pathways (Fig. 6A). These results were supported with the reactome analysis (https://reactome.org), that shows three of pluripotency factors *OCT4, SOX2,* and *NANOG*, bind to the *FOXD3* promoter and further activate its expression (Fig. 8). Previous studies have shown that *FOXD3* is expressed in blastomeres of the inner cell mass^29,30^, considered as a molecular marker of stem cells^31^ and a balanced expression of *FOXD3* is essential to maintain pluripotency^29^. Interestingly, another member of the ZIC family, *ZIC3* is included in the same pluripotency pathway as *FOXD3* (Fig. 8). Mouse ZIC family members, *Zic1, Zic2* and *Zic3* are highly similar to each other and the most extensive homology, of 91%, was observed in their zinc finger domain of *Zic1* and *Zic3*^32^. In addition, mouse *Zic3* was found to bind the *Nanog* promoter which further activates transcription, hence creating a positive feedback loop^33^. Moreover, the involvement of *ZIC3* in enhancing pluripotency and maintaining the pluripotent state of hESCs was previously reported^34^. Hence, we propose that *ZIC1* might also have the same role in inducing and maintaining pluripotency.

Another gene that was exclusively upregulated in A53T-PD2 was *SP8* (7.37 folds) (Supplementary Table S2). This gene is one of the components of Wnt signaling pathway, which is involved in maintaining pluripotency and promoting self-renewal of naïve hESCs^35^. Furthermore, *SP8* was shown to enhance the recruitment of β-catenin, a co-activator that stimulates the expression of Wnt target genes^36^. Of note, *SP8* has recently been identified as a downstream target which is activated by Nanog at day 5 of reprogramming^37^. This upregulation of *SP8* was consistent with our data that showed that the *SP8* gene stays upregulated through reprogramming and self-renewal stages (passage 8). Hence, it is possible that *SP8* is involved in the first stages of reprogramming and is also essential in maintaining the pluripotency in later stages.

In contrast to all other four genes, *PEG3* was the only gene that showed significant down-regulation in A53T-PD2 (− 428 folds, FDR < 0.01) compared to other cell lines. Based on the gene suppression, we put forward the principle in which the knock-down/suppression of *PEG3* would enhance reprogramming efficiency. This hypothesis is supported by the finding that *Peg3* expression levels were decreased in mouse induced pluripotent stem cells (miPSCs)^38^. Moreover, knocking down *Peg3* enhanced the pluripotency state of these PSCs and increased the production of pluripotency markers (*Sox2, Oct4, Nanog, and Rex1*) in mESCs, indicating the crucial role of its inhibition to stimulate self-renewal and maintain pluripotency. *PEG3* inhibition was not only found to play a crucial role in regulating pluripotency, but also in enhancing reprogramming efficiency. Similar to our results (Figs. 5B and 7A), downregulation of *Peg3* increases the number of miPSC colonies derived from mouse embryonic fibroblasts^38^. Through STRING analysis, we predicted Peg3 interactions with many developmental proteins (Peg10, Igf2, Gnas, Mest, Plagl1, Nnat, Ndn and Dlk1) (Fig. 6D). Manipulation of the methylation status of these developmental genes, was found to play a role in regulating human pluripotent stem cells and their differentiated derivatives^39,40^. Hence, these results imply that *PEG3* could be involved in pluripotency regulation by affecting DNA methylation, which plays a role in expressing these developmental proteins.

In summary, we identified five transcription factors that may require selective interactions to mediate pluripotency and reprogramming. In fact, those factors could be used as pluripotency reprogramming factors that work in synergy together and/or with the OSKM proteins in enhancing the efficiency of the reprogrammed cells. Further functional studies are needed to proof this concept and to understand the mechanism of action of these factors to reveal important clues about their role in pluripotency and possibly in iPSC generation.

## Materials and methods

### Study approval

All procedures performed on human samples including the generation of human iPSCs were approved by the Scientific Council and Ethics Committee of Attikon University Hospital (Athens, Greece), which is one of the Mendelian forms of Parkinson’s Disease clinical centers, and by Qatar Biomedical Research Institute Ethics Committee overlooking stem cell research. All experimental methods were performed in accordance with these relevant guidelines and regulations. Written informed consent was obtained from all donors before skin biopsy.

### Human samples

Four dermal fibroblast samples were provided by Drs. Leonidas Stefanis and Kostas Vekrellis from the Biomedical Research Foundation of the Academy of Athens. These samples were isolated from the Greek population after research ethical approval and informed consents were given by sample donors. Among these four samples, one is healthy control (male 45 years old), one represents a sporadic PD patient (female, 69 years old), and two are PD patients with the A53T mutation (A53T-PD1: female, 48 years old; A53T-PD2: male, 43 years old). Ethical approvals were also taken from the IRB committee of the Qatar Biomedical Research Institute, Hamad Bin Khalifa University (QBRI-IRB 2017-002) and from the IRB committe of Qatar University (QU-IRB 1395-E/20).

### Cell culture

Human skin fibroblasts were grown in advanced DMEM/F12 media supplemented with 10% FBS, 1% GlutaMAX and 1% Penicillin–Streptomycin (all were purchased from Thermo Fisher Scientific) and maintained at 37 °C in a 5% CO_2_ incubator. Cells were fed every two days until they became confluent. Cells underwent passage when they reached around 90% confluency by dissociating cells using TrypLE Express reagent (Thermo Fisher Scientific). For stem cell culture, both human embryonic stem cell (hESC) line H1 and human induced pluripotent stem cell (hiPSCs) line IMR90-1 were purchased from WiCell. For cell adherence, feeder-free system was used to grow cells using Matrigel (Corning). Human stem cell colonies were grown and maintained in StemFlex media (Thermo Fisher Scientific) at 37 °C in a 5% CO_2_ incubator. Cells were fed every other day and underwent passage when cell confluency was around 80% (a period of four to five days after a passage). To prepare for cell passage, 35 mm dishes were coated with Matrigel for 30 min, 2-h, or overnight before the passage. At the time of passage, colonies were washed with 1 ml Dulbecco’s Phosphate-Buffered Saline (DPBS) before being dissociated to the appropriate size of cell colonies (usually aggregates are approximately 100 μm in size)^41^. Then, cells were dissociated from the dish using 500 μl of non-enzymatic reagent (ReLeSR; StemCell Technologies), collected by 1 ml StemFlex medium and subsequently centrifuged for 4 min at 800 RPM and 22 °C. Supernatant was aspirated, and the cell pellet was resuspended in 1 ml StemFlex media. Finally, 70 μl of cells were evenly distributed into 2 ml StemFlex media.

### Reprogramming and iPSC generation

Human skin fibroblast cells collected from the subjects mentioned above were used as a source for reprogramming and generating iPSCs. Reprogramming was initiated using CytoTune-iPS 2.0 Sendai Reprogramming kit (Thermo Fisher Scientific) following the manufacturer’s protocol. Briefly, two days prior to transduction, two wells of a 6-well plate were seeded with 1 × 10^5^ fibroblast cells until they reached around 50–80% confluency. On the day of transduction, one well was used to count cells. This well was used as a point of reference, estimating the number of cells in the second well that will be transduced, which should be between 2 × 10^5^ and 3 × 10^5^ cells approximately. The estimated cell count was used for calculating the required volume of each virus to reach the target Multiplicity of Infection (MOI). Transduction of cells was performed with the recommended MOI values of 5:5:3 of KOS (*KLF4*, *OCT4* & *SOX2*), *hc-MYC* and *KLF4* vectors; respectively. However, MOI values of 5:5:6 and 10:10:6 were also used when reprogramming experiments that had very low reprogramming efficiency (healthy control fibroblast cells). Since the titer of each reprogramming vector is lot-dependent; it was ensured the use of the same lot in all our reprogramming experiments, where a titer of 1.5 × 10^8^ (CIU/ml) was used in all the calculations. The calculated volume of each virus vector was added into 1 ml of pre-warmed fibroblast media. Finally, fibroblast media was aspirated from the cells and the reprogramming virus mixture was added to the well that had the cells, which were further incubated at 37 °C with humidified atmosphere of 5% CO_2_ for 24 h. After this period of time, the medium was replaced with fresh and pre-warmed fibroblast medium and cells were cultured for another 6 days with changing medium every other day. On day 7 after transduction, cells were harvested using TrypLE Express reagent (Thermo Fisher Scientific) and counted. Then, cells were seeded into 4 Matrigel coated wells of a 6-well plate with different cell densities (40 × 10^3^, 60 × 10^3^, 80 × 10^3^ and 100 × 10^3^). The remaining cells were harvested for RNA extraction to be used as a positive control when performing quantitative reverse transcription polymerase chain reaction (RT-qPCR) detection of the CytoTune vectors. After cell passage, cells were allowed to grow in fibroblast medium for 24 h; then on day 8 the medium was changed to StemFlex medium, which was replaced every other day thereafter.

### IPSC picking and passaging

Once cell clumps grow to be more stem cell like colonies in size and shape, these colonies were picked and transferred onto prepared Matrigel-coated 6-well plates for further expansion and analysis. Only colonies with distinct borders and bright centers that had tightly packed cells with high nucleus to cytoplasm ratio were manually picked. When colonies became large in size and had defined edges (after 5–6 days after picking), they underwent cell passage using a non-enzymatic dissociation reagent (0.5 mM EDTA in DPBS). Cells were washed with 1 ml DPBS, then 700 μl of dissociation reagent 0.5 mM EDTA was added to the cells and incubated in a 37 °C incubator for 4–5 min. After aspirating the dissociation reagent, detached colonies were collected and part of them were seeded onto a Matrigel pre-coated well of 6-wells plate that was incubated at 37 °C with humidified atmosphere of 5% CO_2_. Picked clones underwent several passages till they reach passage 7, where they were cultured onto 2 wells of a 6-well plate. One well was for freezing colonies using mFreSR media (from StemCell Technologies) and the second well was used for further analysis and characterization experiments.

### Alkaline phosphatase staining

H1 and IMR90-1 cell lines and the 12 cell lines (N = 3 each sample) that were chosen for characterization were also distributed onto 1 well of 4-well plate that was pre-coated with Matrigel. Cells were allowed to grow for 4–5 days before being stained with alkaline phosphatase detection kit (Sigma-Aldrich; Merck Millipore). On the day of staining, alkaline phosphatase staining solution was prepared by mixing Fast Red Violet (FRV) with Naphthol AS-BI phosphate solution and water in a 2:1:1 ratio just before starting the staining procedure. In the beginning, cells were washed with DPBS and then fixed with 4% paraformaldehyde in PBS (Sigma-Aldrich) for 2 min. After fixative solution was aspirated and cells were rinsed with DPBS, staining solution (500 μl) was added to the cells that were then incubated in the dark for 15 min at room temperature (RT) on a microtiter shaker. Sequentially, cells were washed with DPBS and examined under a light microscope for their expression of alkaline phosphatase (red stem cell colonies).

### Immunoblotting

Twenty micrograms (µg) of proteins were separated in 10% SDS-PAGE gels, and followed by a direct transfer into nitrocellulose membrane using Trans-Blot Turbo Blotting System (from Bio-Rad) at 25 V for 14 min. After transfer completion, membranes were blocked at room temperature (RT) for 1 h with 5% low fat milk diluted in 1X TBST (TBS with 0.1% Tween); and then, each membrane was incubated with a protein-specific primary antibody overnight at 4 °C to detect the expression levels of Oct4 (Rabbit, Stemgent), Sox2 (Rabbit, Stemgent), Nanog (Rabbit, Cell Signaling), Klf4 (Rabbit, Abcam), and the loading control Gapdh (Mouse, Invitrogen). All primary antibodies were diluted in the blocking buffer (1:1000 dilution except for Gapdh 1:5000 dilution). The following day, excess primary antibodies were washed off twice with 1X TBST, and membranes were incubated with anti-rabbit and anti- mouse HRP secondary antibodies (GE Healthcare; 1:10,000 dilution in blocking buffer) for 1 h at RT on a rocking platform (List of antibodies used in the study could be found in supplementary information). Proteins were detected using ChemiDoc MP Imaging System (Bio-Rad).

### Immunocytochemistry

In a 4-well plate, cover slips were coated with Matrigel for 30 min, 2 h or overnight; and then incubated with StemFlex medium for 30 min or 2 h to equilibrate the cover slips before cells were seeded on them. H1 and IMR90-1 cell lines and iPSCs from each selected clone that was chosen to be characterized for pluripotency were cultured onto cover slips. When colonies reached the appropriate size, which was usually about 4–5 days post passage, the immunocytochemistry procedures were performed as previously described^42^ using appropriate primary (Oct4 (Rabbit, Stemgent), Sox2 (Rabbit, Stemgent), Nanog (Mouse, Cell Signaling), Klf4 (Rabbit, Abcam), Lin28A (Rabbit, Abcam), TRA-1-60 (Mouse, Millipore), TRA-1-81 (Mouse, Millipore) and Rex1 (Mouse, Santa Cruz) and secondary antibodies dilutions (Supplementary Information). Cells were observed and photographed by a fluorescence microscope (Zeiss Axio Imager 2) using 40X objective.

### RNA and RT-qPCR

Total RNA was extracted from the four primary fibroblast samples, H1and IMR90-1 cell lines, and the 12 generated iPSCs (N = 3 each sample) using TRIzol Reagent (Thermo Fisher Scientific) according to the manufacturer’s protocol. The concentration of RNA was measured using a NanoDrop spectrophotometer. To quantify the expression levels of pluripotency genes and other genes in this study, RT-qPCR was performed using GoTaq 2-Step RT-qPCR System kit (Promega) as instructed by the manufacturer. The generated cDNA was used as a template for the reaction mixture provided by the company and specific primers for each gene (*OCT4, SOX2, NANOG, GAPDH* and *SeV*) were used (Sequences of Forward and Reverse primers are listed in supplementary information). The mixture was added to MicroAmp Fast Optical 96-Well Reaction plate and subjected to thermal cycling (95 °C for 2 min and then PCR reaction: 40 cycles of 95 °C, 15 s and 60 °C, 1 min) using Applied Biosystems 7500 Fast Real-Time PCR System. *GAPDH* was used as a reference gene to normalize the RT-qPCR results of the examined genes.

For validation of RNA-seq findings, standardized RT-qPCR based on TaqMan technology was used according to the manufacturer's protocol using the TaqMan Universal PCR Master Mix (Applied Biosystems). The abundance of each gene was determined using Taqman assay probes, SP8: Hs01941366_s1, GBX2: Hs00230965_m1, PEG3: Hs00300418_s1, ZIC1: Hs00602749_m1, NANOGP8: Hs06596830_s1. The abundance of genes of interest is determined relative to the GAPDH (Hs02786624_g1). Assays were run in triplicates using Applied Biosystems 7500 Fast Dx Real-Time PCR Instrument under default conditions (95 °C for 10 min and then PCR reaction: 40 cycles of 95 °C, 15 s and 60 °C, 1 min).

### RNA sequencing (RNA-Seq)

The transcriptomic sequencing procedure was divided into mRNA library preparation and RNA sequencing. Total RNA samples extracted from fibroblasts, fully characterized iPSC clones, H1 and IMR90-1 cell lines were assessed using a 2100 Bioanalyzer using Agilent RNA Nano 6000. mRNA library preparation was performed using TruSeq Stranded mRNA Sample Prep LS kit (Illumina). The generated libraries were validated by performing quality control analysis and quantification of DNA library templates. Quality control analysis was done using a 2100 Bioanalyzer through the use of a DNA-specific chip (Agilent DNA 1000); while libraries quantification was performed by Qubit assay. The full details of the library preparation can be found in the supplementary information. For cluster generation, indexed DNA libraries were normalized to 10 nM in a diluted cluster template (DCT) plate and pooled together in equal volumes into a pooled DCT plate (PDP). Consequently, RNA sequencing was accomplished using HiSeq 4000 system (Illumina) at the QBRI genomic core facility.

### RNA-Seq analysis

Bcl2fastq Conversion Software was used to both demultiplex the data and convert BCL files generated by Illumina sequencing systems to standard FASTQ file formats for downstream RNA-Seq analysis. FastQC, a quality control tool for high throughput sequence data, was carried out using (https://www.bioinformatics.babraham.ac.uk/projects/fastqc/). The analysis was done according to the typical pipeline of QIAGEN bioinformatics CLC Genomics Workbench 20.0 (https://digitalinsights.qiagen.com). This is a sequential pipeline that starts with 1) pre-processing of raw sequencing reads, 2) mapping of reads to reference, 3) quantification of genes and transcripts, and 4) differential expression analysis.

### Gene ontology molecular analysis

Protein Analysis Through Evolutionary Relationships (PANTHER) 14.1 (GO; http://www.pantherdb.org) is an online classification system that is designed to identify and classify proteins and their genes according to their family/subfamily, molecular function, biological process and pathways in which they are involved in the cell. This software was used to filter the genes involved in our areas of interest: transcriptional regulation and developmental processes.

### Protein–protein interaction analysis

An online software of genes and proteins interactions, Search Tools for the Retrieval Interacting Genes (STRING) 11.0 (https://string-db.org/) was used to construct a predicted interaction between our genes of interest and the universal transcription factors that are well-known to be used in reprogramming (Sox2, Oct4, Nanog and Klf4). Through STRING, the type of interaction between the proteins and the evidence supporting this interaction were also visualized.

### Databases and literature review

Literature evidence was reviewed for the chosen transcriptional factors to confirm their involvement in pluripotency induction using medical search engines like; PubMed, Medline, UpToDate, Scopus, Access Medicine, Genetic Home Reference, and Access Genetics. The following key words ‘the name of the chosen candidate gene’, ‘pluripotency/pluripotent’, ‘reprogramming’, ‘Embryonic stem cells/ESCs’, ‘induced pluripotent stem cells/iPSCs’ were used in our search and the related articles were screened and their findings were documented.

### Statistical analysis

Statistical analyses were performed with GraphPad Prism 8.4.3 (GraphPad Software, Inc., San Diego, CA). The data were expressed as the mean ± SD for each group (N = 3). Statistical significance was tested using the Mann–Whitney U test and defined as *p* < 0.05 or *p* < 0.01.

## Supplementary Information

Supplementary Information.
