# Supplementary material for: Identification of potential transcription factors that enhance human iPSC generation
Source: Sci Rep. 2020 Dec 15;10:21950. doi: 10.1038/s41598-020-78932-9 (PMC7738555; doi:10.1038/s41598-020-78932-9)
Supplement: Supplementary file 1 — Supplementary Information. [file 41598_2020_78932_MOESM1_ESM.pdf]

Identification of potential transcription factors that enhance human iPSC generation

Nuha T. Swaidan, Salam Salloum-Asfar, Freshteh Palangi, Khaoula Errafii, Nada H. Soliman,

Ahmed T. Aboughalia, Abdul Haseeb S. Wali, Sara A. Abdulla, Mohamed M. Emara

Supplementary Figures

Supplementary Fig. 1: A representative Bioanalyzer trace showing the 18S and 28S rRNA peaks from isolated RNA. RNA integrity number (RIN) of more than 8 indicates intact RNA.

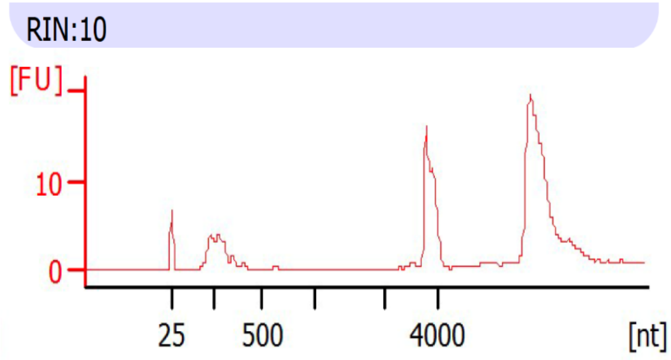

Supplementary Fig. 2: Represents the full-length western blots that are presented in the study.

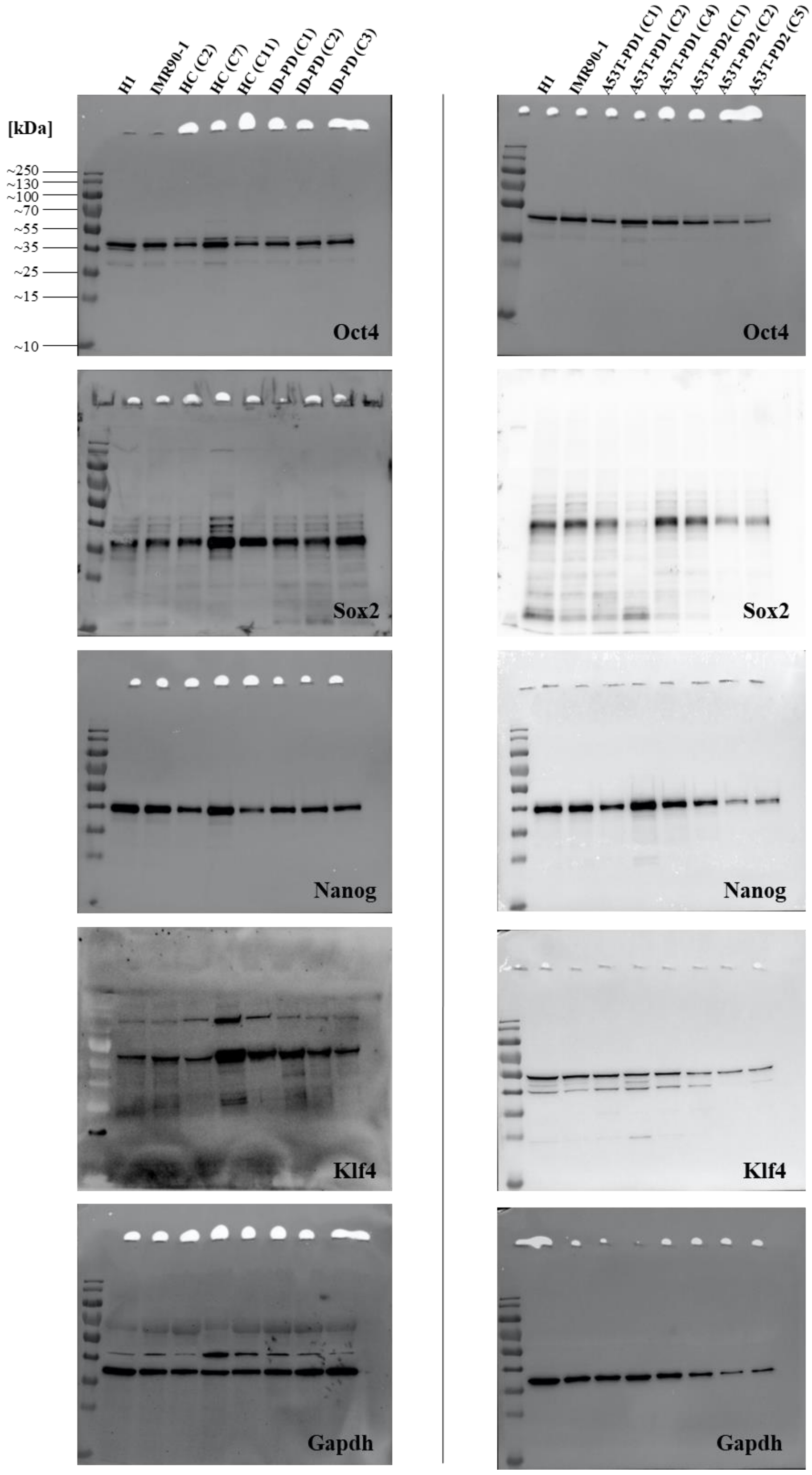

Supplementary Tables

Supplementary Table S1: Percentage of RNA-Seq raw reads that were mapped to reference genome.

Sample ID	Raw reads mapped to reference genome (%)
H1	94.87
IMR90-1	94.65
HC fibroblasts	97.67
HC C2	95.99
HC C7	93.50
HC C11	94.52
ID-PD fibroblasts	97.50
ID-PD C1	84.39
ID-PD C2	94.34
ID-PD C3	94.86
A53T-PD1 fibroblasts	97.74
A53T-PD1 C1	93.97
A53T-PD1 C2	95.69
A53T-PD1 C4	95.43
A53T-PD2 fibroblasts	98.10
A53T-PD2 C1	93.19
A53T-PD2 C2	94.21
A53T-PD2 C5	95.38

Supplementary Table S2: List of differentially expressed genes in A53T-PD2 iPSC clones based on RNA sequencing data.

Name	Fold change	P-value	FDR value
ZNF728	4175.703048	0.000231117	0.043512851
AC003973.3	3367.628971	0	0
CIDEB	1700.171603	0.00107254	0.122032444
FLG	1363.102578	0.001764359	0.169813654
ZNF208	1305.127873	0	0
ZNF729	935.7046356	4.18178E-10	3.20548E-07
ZNF492	728.7081705	7.81208E-12	7.6214E-09
ZNF676	638.0101472	2.09079E-11	1.86978E-08
ZNF99	477.6023122	2.37168E-10	1.88531E-07
ZNF560	435.1961126	0	0
AL049557.1	383.674555	0.011003979	0.527055561
TBC1D3G	358.9605485	0.011903082	0.551783702
ZNF98	341.3960763	9.94982E-13	1.25619E-09
AC026464.6	261.0861926	0.016119328	0.639499345
AC026954.2	234.9932472	0.01967113	0.709582287
ZNF257	233.6975513	0	0
MAS1	198.6456991	0.026321087	0.827129545
TRIM4	182.3098391	0	0
CTSF	159.5441622	0	0
TBC1D3F	152.6619963	0.030774474	0.891411287
POTEC	144.6539291	0.031872069	0.896638364
SDR42E1	135.9065754	1.27336E-11	1.18827E-08
CYP4F2	135.0601366	0.033275687	0.914956806
CSAG1	134.130065	0.036798552	0.944953293
MAGEE2	131.6418566	0.033766397	0.917339976
ZNF572	111.633181	2.78488E-12	2.9886E-09
ZNF662	103.5783804	0	0
ZNF723	82.82155436	3.29336E-06	0.001382606
IFIT2	81.83319498	7.41892E-08	4.30357E-05
PUS7L	81.02486171	0	0
C9orf64	71.77147418	0	0
MT1G	66.47351298	0	0
PABPC3	52.52405677	9.46535E-07	0.000423239
MT1H	52.04243509	3.12195E-13	4.46709E-10
TBC1D3C	45.87910899	1.3237E-06	0.000579806
NKX2-5	41.70828245	0.002484618	0.213011956
IRAK4	41.57684601	1.22125E-15	2.1843E-12
NLRP2	40.91512145	2.79474E-11	2.39934E-08
SLC30A8	40.07003407	0.000654056	0.090567793

PRAC1	36.66180788	0.000780606	0.102159437
MT1F	36.39857639	4.10783E-15	6.78202E-12
FOXC1	31.61899504	5.12826E-05	0.013261177
ZNF528	31.57398319	0	0
PAX7	31.31636481	1.41936E-05	0.004546821
MT1E	30.99982308	1.74616E-12	2.0821E-09
NKAPL	29.06136958	4.51458E-05	0.012265361
IFI44	28.81183477	0.002543517	0.213113482
ZIC1	28.62336484	0.000110968	0.024553722
TLX1	28.42666557	0.001707295	0.165061632
PTPN20	26.56917706	1.10892E-05	0.00379259
FAM24B	25.24101351	9.8555E-05	0.022503033
ISLR2	24.0033989	9.64265E-06	0.003449338
HMX1	23.95065086	9.51262E-05	0.021953684
TBX5	23.3091353	0.00099185	0.11696749
HOXB1	23.17791617	0.001179383	0.128492839
HOXB2	23.05736997	0.002343478	0.206139641
C2CD6	22.78699343	0.004189722	0.293869309
MT1M	21.60579603	0.003206811	0.242921853
PITX2	20.53255195	0.000343156	0.056655041
PRAC2	19.56746273	0.004611187	0.314190208
ANXA2R	19.52410173	0.005370783	0.341761384
PCDHA11	18.69958857	0.007314665	0.414511414
TLE7	18.66632014	0.000702872	0.094939835
HOXB13	18.53174045	0.003542188	0.262158543
BAHCC1	18.0669678	3.68219E-06	0.001491147
TRIM43B	17.65601142	0.014255606	0.6074815
GBX2	16.98551553	1.1752E-07	6.46754E-05
SOX14	16.83644978	0.001116862	0.12485006
SIX3	16.11847697	7.0321E-05	0.017348282
ZIC4	15.75241268	0.000892573	0.109769715
PAX5	15.57242359	0.000432482	0.066779589
TRIM49	15.29740625	0.021113392	0.733263311
PON3	15.17093703	0.013230715	0.596577392
TBX1	14.92933673	0.000999881	0.117064938
SCNN1B	14.89819409	0.00089864	0.109769715
CRISP2	14.61342899	0.013011153	0.587912369
HRNR	14.38198888	0.013469457	0.598429316
MECOM	13.37904335	0.000441122	0.067343655
PAX6	12.60022439	0.005443795	0.344661244
LMX1B	11.97756072	0.00025951	0.048016018
ZFP57	11.79182347	4.24684E-07	0.000202555
GDF7	11.77335021	0.000461676	0.06929331

CCL5	10.93913055	0.010882308	0.524869625
C4orf51	10.85134026	0.001229317	0.130538472
MT2A	10.80644939	1.66772E-08	1.08468E-05
AC005943.1	10.77798729	4.03399E-05	0.011392308
EN2	10.72216904	0.036847257	0.944953293
MYO16	10.58362399	0.00079523	0.103126402
AC006978.2	10.43577088	0.006733958	0.394063402
GAD2	10.39954831	0.002295265	0.203567248
PLD6	10.37566561	0.000710673	0.094939835
TSHZ1	10.34185352	0.000973134	0.115394373
WNT10B	10.32332887	0.000366809	0.059194069
HIST1H3C	10.2752027	0.03298469	0.913000145
MAP3K7CL	10.05051588	0.011371353	0.535226665
TCEAL6	9.981720829	0.011666111	0.544423374
AC010547.4	9.94790133	0.037515955	0.949956294
SP5	9.701002696	0.030358166	0.891238703
PCDHGA3	9.112959607	0.001153106	0.126271011
GDA	9.082759214	4.65183E-14	7.13159E-11
CD36	8.792341451	0.001131368	0.12516776
IRX6	8.765623445	0.023298355	0.769148647
PTPRB	8.663962241	0.000320084	0.054959749
AC139491.7	8.516755894	0.006439591	0.384994245
EOMES	8.441923428	0.004506905	0.309047001
DMBX1	8.28637296	0.000360042	0.058542206
ZNF559	8.278017311	8.84175E-05	0.020937539
HBA2	8.132679506	0.022061696	0.750071031
INSM1	7.921116042	0.00020356	0.03866373
SAMD9	7.791377582	0.010071896	0.498094693
GATA5	7.749397807	0.002083194	0.191075176
OAS2	7.740967886	0.009220759	0.468969537
HIST2H2BF	7.674699871	8.34552E-06	0.003035931
AC008758.1	7.621438392	0.008268341	0.442624232
FOXD1	7.610551097	0.004919565	0.325773147
GSTM5	7.604190602	1.37451E-05	0.004529962
C17orf50	7.585313029	0.020776309	0.72642353
HHLA1	7.568325356	4.11626E-12	4.20702E-09
SP8	7.300236611	1.06618E-05	0.003751368
ZNF705D	7.238115659	0.021522553	0.737920998
KAZALD1	7.204202616	0.00256177	0.213113482
PAX8	7.149063865	0.027318152	0.844855179
PHOX2A	7.095020523	0.008209848	0.442624232
PCDH15	7.023985966	0.00156123	0.153709581
IRX4	6.930996453	0.002970342	0.233967692

CXCL1	6.909426226	0.003849016	0.275371467
ZNF578	6.901241077	5.44732E-06	0.002051157
ZNF502	6.781816052	0.00010962	0.024508117
NKX6-2	6.77975651	0.036841396	0.944953293
IFIT3	6.773152913	0.001109618	0.124689719
NPAS3	6.646288095	0.001519565	0.150932619
CLEC18B	6.598205571	0.029027141	0.871523493
ZNF829	6.583364132	0.000533671	0.07739312
CH25H	6.394255834	0.013585268	0.598429316
C17orf51	6.302781423	0.000698305	0.094939835
GRM6	6.282913549	0.007575633	0.42355763
C1GALT1C1L	6.274810878	0.009283877	0.469952473
C7orf57	6.260682511	0.005303536	0.340807749
UTF1	6.179312086	3.31781E-05	0.009975538
AC020951.1	6.175757289	0.003811659	0.273610856
DND1	6.064959859	3.46532E-05	0.010188515
PRSS3	6.026079658	0.037021147	0.944953293
CTAGE8	6.002952491	0.00030772	0.053695847
PIK3R6	5.989834065	0.008076812	0.44110079
ZNF726	5.938131517	0.001697277	0.164835575
VENTX	5.840426102	0.000143376	0.029841653
LRFN5	5.826216623	1.1309E-05	0.00379259
BHMT2	5.772520748	0.017236275	0.661792796
AL445363.3	5.772303437	0.021509946	0.737920998
MAEL	5.758338134	0.022601483	0.759289111
CNTFR	5.724765097	0.003711991	0.268250717
TSPYL5	5.671627424	0.000370409	0.059328961
CYP4F22	5.661973783	0.00133266	0.13563397
MDGA2	5.501838505	0.000164912	0.032237336
DOK6	5.488660251	0.01073931	0.519139211
MAGEB2	5.30270953	0.022927709	0.767702676
SKOR2	5.266554379	0.019148425	0.696580762
HLA-DQB1	5.24798954	4.13356E-06	0.001642938
ZNF880	5.21713964	4.95665E-08	3.03956E-05
BTK	5.176241044	0.007028182	0.405499645
OLIG3	5.063168312	0.021590323	0.739057609
AC110285.7	5.022261935	0.007009761	0.405499645
CER1	5.015619213	0.0197191	0.710119205
ABHD12B	4.980628629	3.3464E-05	0.009975538
HR	4.935032333	0.033814738	0.917339976
ZNF585B	4.879054295	2.84893E-08	1.79843E-05
TAL1	4.848748134	0.036724338	0.944953293
MT1X	4.823702994	1.64997E-07	8.63738E-05

CCL28	4.814940392	0.00758695	0.42355763
GATA2	4.746616389	0.010494119	0.513064389
SATB2	4.722260408	0.00071217	0.094939835
ZNF559-ZNF177	4.707022597	0.006626997	0.390756129
NFIA	4.668407464	0.008350636	0.442624232
AL139260.3	4.65011305	0.016436087	0.646519874
ZNF630	4.608679353	0.001284044	0.133317986
LTB	4.598178824	0.01851981	0.680395259
AC006064.6	4.576481856	0.005735673	0.359452872
NNAT	4.570818985	0.001219443	0.130436984
PTH1R	4.552838612	0.023582543	0.770398956
PCDHB5	4.550486234	0.000337966	0.056230765
WNT3	4.516249303	0.001066284	0.122032444
CCDC152	4.470144983	2.92486E-05	0.008968044
ZNF439	4.458187001	0.000124588	0.02728602
NANOGP8	4.388448953	2.10626E-09	1.55885E-06
TNRC18	4.373118374	7.44871E-06	0.002756407
ZXDA	4.348090112	0.008545495	0.449206677
BNC1	4.34068214	0.011475863	0.538963802
AC112702.1	4.25577798	0.003693675	0.267975703
TMEM200B	4.213050037	0.000538341	0.077546327
RIMBP2	4.152408853	0.015968123	0.635851243
PCDHB15	4.140126401	0.000399773	0.062630112
Z99129.3	4.065886677	0.001452455	0.144995535
ZNF454	4.007793739	0.007334213	0.414511414
DAPL1	3.998272316	0.01245067	0.56978407
ZNF568	3.986854087	0.000527602	0.077033445
RAB4B-EGLN2	3.981831503	0.037676221	0.949956294
NLRP7	3.961488742	0.00043109	0.066779589
CCDC33	3.92077894	0.013749467	0.599806505
IL9R	3.860073249	0.021472959	0.737920998
PKIB	3.859533584	1.39299E-05	0.004529962
AC100868.1	3.858274312	0.002868064	0.229691264
TFAP2C	3.854419626	0.00044241	0.067343655
ARSE	3.829508031	0.011359708	0.535226665
HHEX	3.820609385	0.024605541	0.791767221
AC004696.2	3.809242953	0.020058639	0.712779074
SNTG1	3.783480936	0.035592065	0.936927096
RBM46	3.777328847	0.002641293	0.217267769
TLL2	3.706404037	0.001221536	0.130436984
ZNF229	3.703993248	0.025955889	0.821668511
XKR9	3.700185462	0.031577722	0.896638364
ZNF850	3.626641545	2.34968E-12	2.65427E-09

ARRB1	3.616738834	0.000319151	0.054959749
VWC2	3.583313764	0.034068457	0.918607144
PDGFA	3.550875028	1.12098E-05	0.00379259
PCDHA1	3.538026175	0.010614226	0.516583085
SLC35E2A	3.523851399	3.34974E-06	0.001382606
LPAR6	3.522165018	0.00321436	0.242921853
ZADH2	3.492064788	0.004118362	0.289811126
CEBPZOS	3.484561501	4.97259E-06	0.001905835
AC008738.7	3.476299926	0.037070804	0.944953293
AL031777.3	3.471540103	0.030987398	0.892923077
ADAMTS9	3.471082713	0.018150608	0.677648256
TGIF2-RAB5IF	3.45978252	0.003442099	0.256520065
IFIT1	3.456784161	0.009443581	0.473569122
COX7A1	3.448273197	0.033933286	0.918423848
AL391005.1	3.433689734	0.023997552	0.776861932
CRIP1	3.428289553	0.031652073	0.896638364
AC135050.2	3.426309121	0.02310321	0.768781685
ZNF441	3.412665483	0.031602853	0.896638364
CYP2B6	3.408679506	0.017162166	0.660128248
MLLT6	3.403702642	4.51143E-05	0.012265361
CAT	3.360839184	0.03838911	0.959191469
BMP7	3.338009187	0.00121058	0.130436984
PCDHA7	3.327526629	0.016677906	0.653207821
ZNF177	3.307007085	0.03921069	0.971617814
POMZP3	3.306942532	4.80758E-05	0.012898126
CDH6	3.262972635	0.003477359	0.258251085
NEFL	3.229241702	0.003693435	0.267975703
CXCL5	3.216989994	0.00471329	0.31912099
PCDHA9	3.213893276	0.007723335	0.428096828
ZNF516	3.203134055	0.000907556	0.109783584
CRLF1	3.189687407	0.001004185	0.117064938
SLC39A4	3.12884262	0.005042668	0.329971891
ZNF717	3.110173426	0.028731303	0.868535151
LHX5	3.060858599	0.014424769	0.611515242
IRX1	3.036741703	0.015586007	0.6255371
FBP1	3.029824644	0.018342826	0.677648256
CPT1A	2.987592845	0.005525765	0.348822062
RAI1	2.979282328	0.008312518	0.442624232
SOCS2	2.936613945	1.24918E-07	6.70277E-05
IGF2_1	2.933072333	0.02600979	0.822162199
HIST2H4B	2.924242895	0.013978105	0.604863054
C17orf107	2.898201211	0.013748349	0.599806505
IER3	2.850696025	0.008172324	0.442624232

TRPC6	2.84859816	0.017575826	0.667663623
ZNF878	2.843253412	0.009380043	0.472591242
DPPA5	2.836218743	0.033418916	0.914956806
CGAS	2.831375322	0.00290706	0.231948797
KCNH5	2.819404942	0.014173979	0.60721779
ZNF382	2.801974335	0.01107785	0.527192679
CDRT4	2.775091191	0.039630568	0.976568185
EPOP	2.766235679	0.008840696	0.459437909
ELAVL4	2.761416387	0.036940259	0.944953293
AP001972.5	2.760369034	0.026799363	0.837255787
FOXB1	2.735071417	0.020001542	0.712779074
DRD4	2.714531435	0.026130016	0.824747855
FAM86B2	2.706405966	0.018358024	0.677648256
ZNF595	2.653745692	0.035180114	0.935651536
DNAJA4	2.653342216	0.034411054	0.922052985
AC011447.7	2.651287072	7.18368E-08	4.28287E-05
FOXI2	2.648229649	0.031062983	0.893706159
ID2	2.636934508	0.00016522	0.032237336
FBN2	2.615859447	8.87721E-05	0.020937539
ZNF737	2.611641362	0.003928923	0.277862646
GDF3	2.583153557	0.009028598	0.463466273
PCDHB3	2.562934584	0.013711464	0.599806505
NETO1	2.556708176	0.000761675	0.10029349
NODAL	2.542658535	0.006006369	0.367278352
ZNF585A	2.474041379	0.000880695	0.109769715
RORB	2.466042099	0.018695788	0.682428056
ZNF283	2.460656043	0.009966818	0.495180123
HES3	2.419600974	0.023098071	0.768781685
NFATC1	2.414386668	0.027700055	0.852978896
CCSAP	2.389455728	0.00058963	0.083809463
PRODH	2.388868305	0.0010746	0.122032444
NEURL1	2.387593136	0.029112915	0.872032857
ZFP3	2.380778815	0.036257345	0.942043625
EIF3CL	2.344362697	0.005339	0.341761384
SULT1A4	2.330155749	0.023216973	0.769148647
H2AFY2	2.325795195	0.003770732	0.271581259
LRRC3B	2.31936138	0.030686549	0.891238703
CCDC169	2.318424514	0.009768007	0.487559827
RIPK4	2.314840731	0.022527546	0.759289111
ZFHX3	2.29848547	0.023477332	0.770398956
ARHGAP28	2.271141219	9.28904E-05	0.021670726
ZNF600	2.268824031	0.018294816	0.677648256
PCDHA3	2.266079116	0.029490438	0.876666593

PEG10	2.244002693	0.000132418	0.028420956
ADAMTS16	2.24158412	0.00904567	0.463466273
ULK4	2.23651951	0.005382069	0.341761384
SLC1A3	2.232575753	0.001081789	0.122202296
NETO2	2.229212052	0.000270816	0.049679771
SALL3	2.201732003	0.000325752	0.05506139
TRABD2B	2.195891558	0.037376368	0.949956294
MN1	2.15901452	0.033470089	0.914956806
AGPAT3	2.153466033	0.014057249	0.60584485
REC8	2.140434105	0.001143797	0.125893902
ALDH1L2	2.125818676	0.00253342	0.213113482
RHOBTB3	2.116422061	0.011954206	0.552780629
COL9A3	2.116233994	4.39981E-06	0.001716964
B3GNT7	2.09312221	0.004756422	0.321028575
STOX2	2.061477118	0.000458936	0.06929331
CHAC1	2.04437265	0.008378745	0.442624232
RGS14	2.037496286	0.035557537	0.936927096
PCDHA10	2.021631009	0.01342184	0.597661746
FAT3	2.014638241	5.09317E-07	0.000237641
ZFP69B	2.010891971	0.020577624	0.724028747
SEPT6	2.010299208	0.007223493	0.412118411
RORA	-2.027373407	0.023236647	0.769148647
TNS1	-2.033440042	0.007092903	0.408136666
ATP1A2	-2.044919017	0.007112173	0.408151253
PTGR2	-2.059136077	0.00082318	0.105648826
ASTN1	-2.084631355	0.015538661	0.6255371
PPFIA4	-2.084753581	0.02792285	0.855750528
NOVA2	-2.090069244	0.006315586	0.379695848
ATP8B3	-2.092953518	0.03775582	0.949956294
KRT7	-2.096137964	0.009047774	0.463466273
HS3ST5	-2.106226226	0.018898119	0.688642325
HAPLN3	-2.10671913	7.47363E-05	0.018228023
SERPINF1	-2.111317921	0.001615419	0.158318482
TCF15	-2.111851434	0.023919849	0.775516196
TCEAL3	-2.119539713	0.008099051	0.441192724
BEX2	-2.150036915	6.32153E-05	0.015776616
CCDC160	-2.15640202	0.037973033	0.953234153
GRIN2A	-2.161961257	0.023213271	0.769148647
SNCG	-2.17797942	0.013737721	0.599806505
NFASC	-2.18191892	0.000614032	0.08557767
SH3BGR	-2.190161745	0.015002554	0.62282363
SULT1A1	-2.208058829	0.028518308	0.863312319
GRPR	-2.209331929	0.014007843	0.604930231

NAAA	-2.210011497	0.015115493	0.624721085
FCGBP	-2.210423601	0.014223588	0.6074815
TCL1B	-2.254417758	0.014854851	0.621500309
SPNS2	-2.258866094	0.035967003	0.93912383
AKR1C3	-2.264585146	0.020977928	0.729739511
BTN3A3	-2.269932597	0.034324937	0.921876223
VIPR1	-2.280702873	0.008926988	0.462801806
DGKB	-2.285964144	0.034348548	0.921876223
BTN3A2	-2.298074064	0.020869198	0.727872869
MUM1L1	-2.300616186	0.027275782	0.844855179
FAM71F1	-2.302638377	0.035226373	0.935722321
POSTN	-2.339015011	0.039384406	0.971617814
CRB2	-2.342330427	0.006738167	0.394063402
ECSCR	-2.344928093	0.037664218	0.949956294
ADAMTS4	-2.359492882	0.0013334	0.13563397
DCX	-2.359795238	0.001322688	0.13563397
TSPAN7	-2.378287828	0.0036957	0.267975703
DACT3	-2.38590912	0.033506781	0.914956806
CD4	-2.393712232	0.006556612	0.387670979
ACOXL	-2.421833974	0.010671244	0.518183052
NPIPA2	-2.430912573	0.000297972	0.052421161
POU3F1	-2.431271707	0.000910662	0.109783584
S100A14	-2.431426836	0.036298284	0.942043625
JPH4	-2.433966357	1.10179E-08	7.38993E-06
MAPK8IP2	-2.437629883	0.01748814	0.667480651
CCDC169-SOHLH2	-2.442985849	0.032325846	0.902223195
FRRS1L	-2.465721232	0.025146849	0.803164912
SYP	-2.479064559	0.007207075	0.412118411
KLK6	-2.501139925	0.031427313	0.896638364
SMIM10L2A	-2.529924375	0.003227758	0.243078518
BCL2L14	-2.530820592	0.026266701	0.827129545
BTG2	-2.545265215	0.002529927	0.213113482
DNM3	-2.545678048	0.017853091	0.673428619
SCUBE1	-2.547067514	0.004712191	0.31912099
CHST13	-2.54944047	0.033715551	0.917157009
LRFN2	-2.5641843	0.017434959	0.667480651
RASA4	-2.574999906	0.001525993	0.150932619
NRXN2	-2.579000009	0.00197862	0.187079854
SORCS3	-2.584543396	0.012484841	0.57013223
AL022318.4	-2.588413881	0.035636427	0.936927096
AC148477.5	-2.627925683	0.029827785	0.880596636
VIL1	-2.62867303	0.005936418	0.365437729
TMEM151B	-2.630918415	0.00014599	0.029841653

PAPLN	-2.635858918	1.67425E-06	0.000718689
TLR5	-2.643428166	0.015231345	0.625067621
CACNA1E	-2.651251454	0.014960215	0.622269561
RAI2	-2.653528564	0.029403372	0.875290663
PKDCC	-2.655270007	0.000601369	0.084498821
LHFPL6	-2.656687639	0.003935622	0.277862646
AC013717.1	-2.664656417	0.034361505	0.921876223
TNS4	-2.664852608	0.033561765	0.915293724
FAM107A	-2.667357913	0.035516466	0.936927096
KCNJ6	-2.679687872	0.031401462	0.896638364
PAMR1	-2.706612485	0.002309284	0.203967738
NPIPA7	-2.71648715	0.007758918	0.428096828
SEZ6	-2.720135075	0.020464243	0.722407968
CD79B	-2.743692125	0.014647178	0.616416454
MPZL2	-2.757224174	0.015211421	0.625067621
PKNOX2	-2.761510532	0.003684794	0.267975703
LCN10	-2.762759026	0.023578039	0.770398956
MAGED4	-2.76721943	0.022605681	0.759289111
ARHGAP30	-2.790057065	0.036771356	0.944953293
AC008012.1	-2.801789475	0.006152203	0.371956994
CAVIN3	-2.817543558	0.000142279	0.029841653
C16orf89	-2.845601546	0.028167009	0.859870393
SCARA5	-2.867427763	0.036399316	0.943524771
WFIKKN1	-2.870577992	0.002222328	0.199572452
SCG2	-2.885006754	0.010490214	0.513064389
SBK2	-2.888154128	0.011758947	0.547466985
SUSD4	-2.894589369	0.004221846	0.294543135
CTNNA3	-2.900282156	0.027898143	0.855750528
KCNK2	-2.905409079	0.01518641	0.625067621
COL1A1	-2.912157929	1.8837E-07	9.40231E-05
S100A4	-2.925366763	0.000915588	0.109783584
SNAP91	-2.936540256	0.021409594	0.737582868
PAQR6	-2.950583229	0.002864354	0.229691264
CCKBR	-2.984885234	0.000145914	0.029841653
APLN	-2.98782591	0.015854624	0.632505204
EIF5AL1	-3.046915543	0.037665764	0.949956294
P2RX1	-3.071907936	0.019863459	0.71054903
CYP1B1	-3.076965168	0.000826958	0.105648826
MYOF	-3.080163201	0.004881266	0.324354846
EEF1AKMT4-ECE2	-3.085872984	0.032614573	0.907920329
C11orf86	-3.087137565	0.03540573	0.936197678
SBK3	-3.096684205	0.026485866	0.831090863
EDN1	-3.098154047	0.028790892	0.868701937

CNTN2	-3.100649242	0.017539259	0.667480651
SPTLC3	-3.101024571	0.018346463	0.677648256
PTPRH	-3.1082461	0.02578925	0.818892251
TEX11	-3.123141894	0.020343691	0.719335485
KLK7	-3.128180688	0.007353189	0.414511414
APELA	-3.138092238	7.46601E-07	0.000340942
ALOX15B	-3.152111088	0.023845893	0.774288035
FAM43B	-3.18222018	0.004948145	0.325773147
MYLPF	-3.184412045	0.038925249	0.968079524
SERPINF2	-3.186624567	0.004982501	0.327031856
RGS9	-3.196507313	0.015330418	0.6255371
DPEP1	-3.214185827	0.008389497	0.442624232
VAV1	-3.243947219	0.000580046	0.082996879
STC1	-3.244192479	5.86511E-09	4.06074E-06
CD34	-3.251367806	0.033687763	0.917157009
GABRA1	-3.266594425	0.035872029	0.938928493
ACTG2	-3.266641093	0.016859812	0.657409538
SCRT2	-3.280895851	0.018360046	0.677648256
CUZD1	-3.281096325	0.00600141	0.367278352
CPNE5	-3.287705188	0.011214543	0.531341596
MX2	-3.294723131	0.017088422	0.659656117
LHX1	-3.29905708	0.016446902	0.646519874
LRRC15	-3.314934621	0.033120993	0.914898166
TTPA	-3.3199244	0.018134578	0.677648256
DOK7	-3.34120275	0.029362637	0.875290663
HKDC1	-3.351864604	0.036494633	0.944854415
PRELP	-3.366675425	0.017715481	0.670792215
AL355315.1	-3.408125177	0.032518053	0.906409054
C6orf118	-3.437942806	0.01513558	0.624721085
BTBD17	-3.454220749	0.002449901	0.212025084
CLEC14A	-3.454871668	0.038560277	0.960697171
PKP3	-3.456243216	6.0153E-05	0.01518898
MS4A15	-3.458142738	0.037724688	0.949956294
ABCC3	-3.468117716	0.023567626	0.770398956
PDYN	-3.471915289	0.014727565	0.617378356
PARVG	-3.51280413	0.014137899	0.606883459
KCNN4	-3.516380722	0.013606369	0.598429316
SMIM24	-3.517093545	0.000395997	0.062494662
AL590560.1	-3.522334976	0.031875091	0.896638364
HLA-G	-3.537344306	0.00319852	0.242921853
TRPC4	-3.548259039	0.01386408	0.602357776
OPTC	-3.604680145	0.002524268	0.213113482
SMOC1	-3.617770395	0.006688812	0.393320451

ANKS4B	-3.621434498	0.036978431	0.944953293
TIE1	-3.645352095	0.002023885	0.187710464
TNNT2	-3.678933778	0.006147609	0.371956994
CACNA1S	-3.702282774	0.026317602	0.827129545
IQSEC3	-3.704884113	0.00194529	0.184742343
EIF4E1B	-3.757561348	0.002974325	0.233967692
RD3	-3.75767689	0.010040647	0.497696077
MB	-3.761402748	0.013816138	0.601492443
KIF19	-3.766169524	0.002989935	0.234207938
MAGEA2B	-3.776460292	0.030649538	0.891238703
ROPN1	-3.784850065	0.023787236	0.774288035
FRMPD2	-3.839699231	0.029689306	0.877715657
IQCN	-3.855734481	0.030994162	0.892923077
HGF	-3.885579056	0.006505849	0.386044498
GALNT9	-3.903154461	0.007756437	0.428096828
CHRND	-3.942618734	0.021252283	0.734521326
ALAS2	-3.982584074	0.021184565	0.7334747
SHOX2	-3.986039814	0.039977041	0.982181997
GJA5	-4.019567829	0.001385734	0.139756917
JPH2	-4.027356232	0.001273283	0.13330965
TRIM50	-4.03526151	0.029306549	0.875290663
AC008982.1	-4.037842473	0.035002144	0.932073227
AVPR1B	-4.054249193	0.021746902	0.740878995
GLIS1	-4.057196074	0.03579472	0.938048929
GJB5	-4.112214136	0.006906531	0.402812183
AIPL1	-4.125846521	0.001988991	0.187126377
PCDH8	-4.238207627	0.006119343	0.371956994
ANGPT4	-4.239367319	0.008315035	0.442624232
APBB1IP	-4.278430007	0.01978643	0.71054903
KCNT1	-4.29630005	0.011015851	0.527055561
F10	-4.48421649	0.000602354	0.084498821
GNA15	-4.504343013	0.004226776	0.294543135
C7	-4.540060133	0.03706798	0.944953293
MATN4	-4.593809205	0.000284437	0.051301476
AMH	-4.612551898	0.000186196	0.036002864
ADRB3	-4.672852999	0.014445242	0.611515242
MAGEA4	-4.689923076	0.000891437	0.109769715
CCDC177	-4.749294083	0.002182506	0.196819829
KLK5	-4.8009389	0.000673321	0.092637765
ASCL5	-4.819166769	0.002443297	0.212025084
MAP1LC3C	-4.845711859	0.000283755	0.051301476
FGFBP1	-4.87055288	0.019961078	0.712779074
ART3	-5.063928433	0.003059139	0.238757463

LAPTM5	-5.072218587	0.006495874	0.386044498
PTPN7	-5.136905212	0.010230799	0.504032266
TTLL10	-5.2246248	0.006928898	0.4030215
LRRC24	-5.466102991	0.020568643	0.724028747
RNASE1	-5.61711081	0.000797604	0.103126402
FETUB	-5.637253084	0.032081134	0.900677574
NNMT	-5.63870593	0.032315881	0.902223195
CYP1A1	-5.723195526	0.001386955	0.139756917
RBPJL	-5.771544748	0.00319293	0.242921853
AC011472.5	-5.935466634	0.022086609	0.750071031
ADRA2A	-6.041439523	0.000147472	0.029860255
NPIPB7	-6.061733725	0.006991557	0.405499645
ITIH3	-6.232362453	0.031733572	0.896638364
CYP2A7	-6.250183739	0.023037821	0.768781685
VSTM1	-6.274866704	0.008369767	0.442624232
CD300A	-6.300477131	0.000157195	0.031239535
XPNPEP2	-6.522860639	0.003632272	0.267901919
FMO1	-6.864059208	0.01495981	0.622269561
FMOD	-6.910304923	0.000131714	0.028420956
AL079342.3	-7.069010671	0.027281144	0.844855179
NPTX1	-7.092583246	1.60546E-10	1.32531E-07
H1FOO	-7.097144514	0.024094065	0.778536938
OR52A1	-7.209967166	0.000144456	0.029841653
APOBEC3H	-7.227415374	0.000834797	0.106019242
AL136454.1	-7.962908419	0.015510941	0.6255371
AL360181.3	-8.219349568	0.017720691	0.670792215
AC097637.1	-8.491493828	0.018412607	0.677855541
MAGEA2	-8.570094938	0.001128407	0.12516776
OR2T8	-8.59084872	0.015760842	0.629934753
CD3D	-8.715782015	0.016842037	0.657409538
SOHLH1	-9.127715268	0.036931132	0.944953293
FAM71E2	-9.129836912	0.030201571	0.89040703
PGM5	-9.132344893	0.034053541	0.918607144
TRIM29	-9.265995054	0.001833533	0.17568358
PDE11A_2	-9.691540091	0.010738507	0.519139211
FKBP1C	-9.693421222	0.008393424	0.442624232
XCR1	-9.866950114	0.005931933	0.365437729
RGPD1	-10.13223919	2.75293E-05	0.008563199
FXYD2	-10.56265125	0.005640892	0.355045323
PASD1	-10.60368151	0.021698103	0.740391711
TRAC	-10.76522878	3.79163E-05	0.01099727
AL031847.2	-11.02141457	4.47123E-05	0.012265361
SMYD1	-11.06261309	0.000257225	0.048007102

SMIM38	-11.16062787	0.001902537	0.181485089
MYT1L	-11.41741319	0.000327119	0.05506139
UTS2	-11.46611844	0.002261736	0.202068153
OR2A12	-11.78827924	0.019806452	0.71054903
BPIFB4	-11.90012113	0.019369031	0.703413709
NPIPA8	-12.10638583	1.84645E-07	9.40231E-05
HBA1	-13.15830563	0.002975967	0.233967692
DHRS2	-14.20243995	7.79787E-08	4.40436E-05
CES1	-14.24797379	3.50975E-09	2.51099E-06
CHL1	-16.78225136	0.01410681	0.606762471
RGPD2	-18.00157709	3.94679E-05	0.011294653
AC011479.1	-19.43644422	0.003268291	0.245270389
PRR33	-19.6983416	0.00626658	0.377807911
TRBC1	-20.66642851	4.92906E-05	0.012954965
TPSD1	-22.33091243	0.002803785	0.226231743
ZIM2	-25.0081157	0.004836899	0.324354846
AC068631.2	-30.00048717	0.000108762	0.024508117
AF241726.2	-35.43853693	0.039956053	0.982181997
FGF5	-35.4942535	0.028964646	0.871523493
VCY1B	-36.02747468	0.036633429	0.944953293
CSAG2	-40.17711067	0.030775556	0.891411287
RHOXF2	-40.41504785	0.02341205	0.769514288
ARL2-SNX15	-41.43600979	0.031851834	0.896638364
SAGE1	-41.43600979	0.031826936	0.896638364
AC127029.3	-43.20887279	0.023830718	0.774288035
APOA2	-45.58300745	0.02032948	0.719335485
KCNC2	-50.25809061	0.016994341	0.659583272
AC009133.6	-50.28352967	0.016179254	0.640210055
SIRPB1	-84.78232928	0.006511117	0.386044498
AC010531.1	-94.69173801	0.005380715	0.341761384
SPATA31A5	-95.10557756	0.008560105	0.449206677
CT45A10	-122.6583775	0.004600245	0.314190208
OR2A4	-134.0066348	0.002558288	0.213113482
MTRNR2L1	-153.5505327	0.003112468	0.242039512
AC139530.2	-160.5113387	0.003161819	0.242921853
AC008695.1	-176.880139	2.31677E-07	0.000113011
CXCL11	-183.3630061	0.00133062	0.13563397
AC048338.1	-243.6904673	0.001404179	0.140831284
TBC1D3D	-427.4234094	0.000377308	0.059986427
PEG3	-436.0755845	8.27338E-13	1.10982E-09
AL035078.4	-1200.023002	2.27593E-05	0.007183581

Supplementary Table S3: List of the 91 differentially expressed genes that showed an FDR ≤ 0.05 in A53T-PD2 iPSC clones.

Name	Chromosome	Region	HC vs. IMR90-1 Fold change	HC vs. IMR90-1 P-value	HC vs. IMR90-1 FDR	A53T-PD1 vs. IMR90-1 - Fold change	A53T-PD1 vs. IMR90-1 - P-value	A53T-PD1 vs. IMR90-1 - FDR	A53T-PD2 vs. IMR90-1- Fold change	A53T-PD2 vs. IMR90-1-P-value	A53T-PD2 vs. IMR90-1 - FDR	ID-PD vs. IMR90-1-Fold change	ID-PD vs. IMR90-1 - P-value	ID-PD vs. IMR90-1 - FDR
AC003973.3	19	complement (21965708..21968529)	2843.161565	1.941E-12	5.95139E-09	1147.342462	4.5209E-10	1.61719E-06	3215.13364	8.8396E-13	2.872E-09	4154.05977	1.65E-13	4.183E-10
ZNF208	19	complement (21932958..22010949)	1111.806989	0	0	505.9070063	3.1641E-14	1.6978E-10	1319.874271	0	0	1591.40892	0	0
ZNF729	19	22286408..22317176	1396.388334	1.84284E-08	2.32664E-05	705.4693923	3.4844E-07	0.000415472	937.4849206	1.0591E-07	8.1181E-05	2011.59522	3.43E-09	4.325E-06
ZNF492	19	22634324..22667670	433.257223	1.49313E-07	0.000160235	230.8766142	2.5169E-06	0.002250821	778.5546218	8.35E-09	9.4324E-06	727.966473	1.18E-08	1.054E-05
ZNF676	19	complement (22179091..22215775)	883.4225628	2.41884E-09	3.24473E-06	567.7359744	2.4397E-08	4.76023E-05	642.7013208	1.295E-08	1.3898E-05	1083.42911	7.93E-10	1.135E-06
ZNF99	19	complement (22752183..22784107)	513.8891199	2.23925E-07	0.000218459	223.7016485	7.2494E-06	0.005019139	511.8680986	2.2819E-07	0.00016848	597.95419	1.13E-07	8.117E-05
ZNF560	19	complement (9466507..9498607)	359.7193489	2.37921E-13	1.0213E-09	86.10954242	3.0472E-08	5.45022E-05	472.7234218	1.7764E-14	7.6252E-11	519.917832	6.99E-15	2.502E-11
ZNF98	19	complement (22391019..22532485)	359.8925954	2.18791E-08	2.60884E-05	178.1744126	8.3843E-07	0.000782401	342.4279315	2.8762E-08	2.5722E-05	517.869444	2.81E-09	3.765E-06
ZNF257	19	22052452..22091480	173.5621143	2.81642E-10	5.0374E-07	65.25192404	3.2674E-07	0.000412516	247.6077561	1.5438E-11	3.0123E-08	209.489616	6.22E-11	1.027E-07
CTSF	11	complement (66563463..66568841)	159.1791615	9.99201E-16	5.36146E-12	126.0839586	1.8208E-14	1.30264E-10	170.0399643	4.4409E-16	2.3829E-12	106.336695	1.45E-13	4.183E-10
TRIM4	7	complement (99876958..99919600)	170.6607023	1.02129E-12	3.65E-09	83.14678133	9.0322E-10	2.76939E-06	165.7032594	1.3813E-12	3.2942E-09	142.28385	6.29E-12	1.124E-08
SDR42E1	16	complement (81988855..82011488)	190.9818592	7.2663E-12	1.94946E-08	50.82854126	3.0988E-07	0.000412516	137.6966645	1.33E-10	2.3788E-07	275.624889	2.28E-13	4.899E-10
ZNF572	8	124973298..124979389	116.3044764	2.52341E-10	4.92363E-07	70.47116163	1.5741E-08	3.37853E-05	113.2503252	3.2009E-10	4.6067E-07	109.231335	4.4E-10	6.753E-07
ZNF662	3	42905731..42917641	77.06535915	2.03436E-09	2.91089E-06	36.00059739	7.9238E-07	0.000773034	97.96635854	2.5057E-10	4.137E-07	62.2009302	1.23E-08	1.057E-05
ZNF723	19	22832321..22858667	179.5592188	5.12894E-07	0.000423394	1.327694786	0.79843819	0.999973169	83.49949301	1.8895E-05	0.00664824	111.53596	5.16E-06	0.001978
IFIT2	10	89283694..89309276	86.28483122	6.5207E-05	0.022943254	18.34314339	0.00928188	0.784319071	82.17324294	7.8467E-05	0.02105184	4.92870532	0.156662	0.9999755
PUS7L	12	complement (43718993..43758817)	64.87641838	0	0	63.36108878	0	0	77.10773006	0	0	63.0298543	0	0
C9orf64	9	complement (83938311..83956986)	57.4828941	8.94121E-11	1.91905E-07	25.5858799	2.2077E-07	0.000338453	74.94734419	4.8808E-12	1.0476E-08	75.7130283	4.38E-12	8.54E-09
MT1G	16	complement (56666731..56668065)	75.81638832	1.66062E-07	0.000169724	-1.15061573	0.86916313	0.999973169	68.11059486	3.3303E-07	0.00022337	22.1118005	0.000184	0.0346832
PABPC3	13	25095868..25099254	64.65500229	4.15406E-07	0.000383939	41.16298253	6.6567E-06	0.004762412	52.89844314	1.4854E-06	0.000797	73.7786314	1.79E-07	0.0001203
MT1H	16	56669814..56671129	61.55136629	2.58583E-06	0.001585704	1.340200278	0.74649255	0.999973169	50.90122833	7.352E-06	0.00309403	13.9704568	0.002705	0.2015805
IRAK4	12	43758944..43789543	33.46484061	0	0	33.05234293	0	0	42.18239902	0	0	37.3167174	0	0
NLRP2	19	54953130..55001142	24.77811617	2.3991E-05	0.010727497	14.12651557	0.00049352	0.121752549	41.22025803	9.8801E-07	0.00055226	22.188918	4.53E-05	0.0110496

MT1F	16	56657694..56660698	22.38453129	0.000108218	0.032259451	-1.6179178	0.56437345	0.999973169	36.64125419	7.2445E-06	0.00309403	9.53998975	0.00506	0.2966417
ZNF528	19	52397849..52418412	21.97253297	3.12741E-10	5.16335E-07	6.424527629	0.00016282	0.052157233	33.26849834	9.367E-13	2.872E-09	15.8078312	1.98E-08	1.571E-05
FOXC1	6	1609972..1613897	-2.16735019	0.38518134	0.99997305	-1.7024338	0.54928322	0.999973169	31.93030327	4.3691E-05	0.01267226	-2.1378642	0.401726	0.9999755
PAX7	1	18631006..18748866	-1.137103618	0.867683567	0.99997305	1.290191685	0.74037301	0.999973169	31.64414743	5.3688E-06	0.00245172	-3.0311479	0.167246	0.9999755
MT1E	16	56625475..56627112	30.81486003	2.5985E-05	0.011381948	-1.36401616	0.71595323	0.999973169	30.78601264	2.6202E-05	0.00815041	10.4739047	0.004059	0.2624217
ZIC1	3	147393422..147510293	-5.375334134	0.039233129	0.703811052	-3.16657426	0.15462706	0.999973169	29.00707118	2.3082E-05	0.00750627	-14.226322	0.002186	0.1773757
FAM24B	10	complement (122849078..122879641)	18.36913438	0.000591664	0.096203611	15.3655869	0.00131064	0.22521866	26.73735849	0.00010255	0.02589394	38.6771708	1.52E-05	0.0048597
PTPN20	10	46911396..47002488	43.87080818	1.03917E-06	0.000810754	24.45941647	3.8228E-05	0.018647611	26.16660398	2.5929E-05	0.00815041	44.1467263	1.02E-06	0.0004974
ISLR2	15	74100311..74138540	-1.019025628	0.978410941	0.99997305	1.585451039	0.50616047	0.999973169	24.21533568	3.2283E-06	0.00153978	-1.344993	0.674819	0.9999755
BAHCC1	17	81395475..81466332	-1.099989886	0.873489469	0.99997305	1.626739528	0.41498137	0.999973169	18.26767246	1.0035E-06	0.00055226	-2.5066392	0.131072	0.9999755
GBX2	2	complement (236165236..236168369)	1.241968034	0.670425778	0.99997305	1.457034969	0.4598506	0.999973169	17.15594455	1.7838E-08	1.7403E-05	1.00324574	0.994956	0.9999755
PAX5	9	complement (36833275..37034185)	-2.613428489	0.202781569	0.985882281	-1.44243767	0.62333891	0.999973169	15.73408176	0.00015992	0.03538441	-4.4412862	0.057932	0.9504012
LMX1B	9	126614443..126701032	-1.055012888	0.93576581	0.99997305	1.793330009	0.3774426	0.999973169	12.1055455	0.00014993	0.03426669	1.06090763	0.929277	0.9999755
ZFP57	6	complement (29672392..29681110)	1.874873101	0.143142222	0.935809173	1.990013906	0.10941862	0.999973169	11.91608468	5.6856E-09	6.7794E-06	49.5923356	0	0
GDF7	2	20666664..20679245	1.941786627	0.318732159	0.99997305	1.321626717	0.68120593	0.999973169	11.85952282	0.00014628	0.03412648	1.86370002	0.355016	0.9999755
AC005943.1	19	complement (1578339..1605445)	6.764554407	0.001419952	0.168378066	6.422473624	0.0019284	0.292943673	10.89262796	6.6175E-05	0.01820912	1.53412833	0.483138	0.9999755
WNT10B	12	complement (48965340..48971763)	5.521122425	0.006099591	0.370865492	7.450088604	0.00123625	0.217489554	10.42284642	0.00015008	0.03426669	6.2165779	0.003503	0.2376865
GDA	9	72114595..72257193	4.617232453	0.000513807	0.08843273	2.615404053	0.02919331	0.999973169	9.190588204	4.6985E-07	0.0002966	2.47015072	0.040322	0.8493005
PTPRB	12	complement (70515866..70637440)	-1.493860712	0.475863103	0.99997305	1.227056353	0.71505085	0.999973169	8.755203473	9.6652E-05	0.02469576	1.46974822	0.491895	0.9999755
ZNF559	19	9323772..9351162	7.523884086	2.07395E-06	0.001343991	5.328046037	8.4148E-05	0.033445829	8.355266783	5.9658E-07	0.00035568	7.9015749	1.17E-06	0.0005598
HIST2H2BF	1	complement (149782689..149812373)	4.795306151	0.00048722	0.086423089	6.176790834	5.0201E-05	0.021989028	7.809215216	4.4543E-06	0.00207834	4.06821215	0.001952	0.1636139
GSTM5	1	109711780..109775428	6.055626949	0.000860651	0.124811887	5.347346281	0.001947	0.292943673	7.803070194	0.000143	0.03372845	7.84059771	0.000139	0.0279149
SP8	7	complement (20782283..20786886)	-1.129507539	0.776316855	0.99997305	1.155259962	0.73624468	0.999973169	7.375965407	2.9082E-06	0.00141863	-1.6810019	0.227427	0.9999755
ZNF578	19	52453576..52512154	4.760595684	0.000450284	0.082602092	1.413448749	0.44377575	0.999973169	7.017550007	1.1544E-05	0.0045049	1.5646178	0.322216	0.9999755
ZNF502	3	44712643..44723831	6.229779911	2.11984E-05	0.009680469	4.598378433	0.00039735	0.105914545	6.813731672	8.2169E-06	0.00332752	4.73895441	0.000306	0.0497163
C17orf51	17	complement (21428381..21574517)	5.259219855	4.91807E-05	0.01919209	4.377952018	0.00030755	0.084626172	6.361404506	6.041E-06	0.00264609	5.74879782	1.91E-05	0.005602
CTAGE8	7	complement (144266701..144269288)	4.475574455	0.001381774	0.165681682	3.801546033	0.00445889	0.525829956	6.052465128	0.00012036	0.02902595	4.58259237	0.001184	0.1190289

VENTX	10	133237404..133241929	1.647165985	0.25837444	0.99997305	1.37350168	0.47290785	0.999973169	5.895714192	5.6492E-05	0.01574666	1.168196	0.725576	0.9999755
ZNF880	19	52369917..52385795	3.635909346	1.44433E-05	0.007045354	2.252269616	0.00650351	0.667870367	5.268390447	2.3256E-08	2.1702E-05	3.0511498	0.000183	0.0346832
ZNF630	X	complement (47983356..48071658)	4.620105269	0.000376264	0.072754546	3.603359781	0.00304639	0.399149536	5.070716318	0.00016336	0.03577684	4.219495	0.000872	0.1001224
ZNF585B	19	complement (37181579..37218153)	4.597120814	1.63479E-11	3.89862E-08	3.970367047	1.2305E-09	3.30124E-06	4.995881178	1.2508E-12	3.2942E-09	3.79769417	4.31E-09	4.874E-06
CCDC152	5	42756801..42802360	4.400788377	4.29322E-07	0.000383939	3.36032194	3.6161E-05	0.018315259	4.504369588	2.8461E-07	0.00019705	4.42794579	3.92E-07	0.0002142
NANOGP8	15	complement (35084193..35085110)	2.550860767	0.001880909	0.198866702	1.488325411	0.18766594	0.999973169	4.422695594	7.8918E-07	0.00045779	2.35607917	0.004479	0.2788152
TNRC18	7	complement (5306790..5425414)	1.634888594	0.118968926	0.905102764	1.595314714	0.13875669	0.999973169	4.414220539	2.3897E-06	0.0011928	1.61345139	0.129646	0.9999755
PCDHB15	5	141245349..141249365	2.946337403	0.001806281	0.193841076	2.467697213	0.00919571	0.783204757	4.16477863	3.7532E-05	0.01134589	3.40358999	0.000408	0.0588041
PKIB	6	122471917..122726373	3.74502518	0.000174458	0.046562994	2.855493863	0.00286779	0.382306495	3.901470819	0.00010909	0.02722569	4.91044289	6.08E-06	0.0022023
ZNF850	19	complement (36714383..36772825)	3.209650516	8.22964E-08	9.29646E-05	2.076861802	0.00079494	0.156994174	3.664372592	2.3603E-09	3.1662E-06	3.5977452	4E-09	4.773E-06
PDGFA	7	complement (497258..520296)	1.645386009	0.047546708	0.740858034	1.362809886	0.21847215	0.999973169	3.582568975	3.7338E-07	0.00024284	1.28640669	0.31702	0.9999755
SLC35E2A	1	complement (1724838..1745999)	1.503215451	0.183413389	0.968096399	2.647693044	0.00147454	0.243446455	3.557668068	3.3889E-05	0.0103909	-1.0329474	0.915906	0.9999755
CEBPZOS	2	37196488..37216193	2.394120408	0.000336272	0.069680256	1.962726858	0.00563748	0.600117753	3.519122668	2.3549E-07	0.00016848	2.84033736	1.81E-05	0.0054798
MLLT6	17	38705542..38729803	1.47760547	0.20156762	0.984804422	1.910003806	0.03425236	0.999973169	3.434409854	5.339E-05	0.01507787	-1.5991361	0.126432	0.9999755
SOCS2	12	93569814..93583487	2.608184148	7.64676E-05	0.02449588	1.95946367	0.00555522	0.596158302	2.952336999	7.958E-06	0.00328467	2.11289557	0.002048	0.1683935
AC011447.7	19	complement (20220597..20222186)	2.183417689	1.22182E-05	0.0060986	1.647939693	0.00525024	0.586905288	2.674363997	3.5781E-08	3.0719E-05	1.79008075	0.001145	0.1158892
ZNF585A	19	complement (37106734..37172741)	2.131924748	0.000971456	0.138081838	1.813210773	0.00964146	0.789827317	2.49371319	6.846E-05	0.01859943	2.16576812	0.000774	0.0912576
ARHGAP28	18	6729718..6915716	1.8543541	0.001220177	0.158866948	1.517179216	0.02934248	0.999973169	2.288956976	1.4396E-05	0.00542089	1.54988847	0.022108	0.6529541
PEG10	7	94656325..94669695	1.510514076	0.011225186	0.478470145	1.235036472	0.19450154	0.999973169	2.264411186	5.0259E-07	0.0003082	1.58599774	0.004585	0.2811897
NETO2	16	complement (47077703..47143997)	2.325478117	9.25005E-06	0.004726995	1.589802429	0.01512833	0.950382921	2.244294919	2.1964E-05	0.00725245	1.17747092	0.39362	0.9999755
STOX2	4	183797692..184023526	1.461335562	0.026820501	0.637508359	1.416351327	0.0423711	0.999973169	2.076989105	1.9643E-05	0.00665972	1.15272091	0.408132	0.9999755
FAT3	11	92352096..92896470	1.588830685	0.000411676	0.077507047	1.643418896	0.00015123	0.049178425	2.02923923	6.663E-08	5.2966E-05	-1.1283258	0.358411	0.9999755
HAPLN3	15	complement (88877288..88895626)	-1.573302718	0.008796332	0.428997266	-1.25066922	0.19590901	0.999973169	-2.09548553	1.9494E-05	0.00665972	-1.7503164	0.00123	0.1199607
BEX2	X	complement (103309346..103311046)	-1.441665154	0.033517754	0.676119886	-1.36720863	0.06921618	0.999973169	-2.13890438	1.0375E-05	0.00412387	-1.2692075	0.166174	0.9999755
NPIPA2	16	14748066..14765413	-2.397243462	5.75661E-05	0.021676168	-1.55666534	0.0413415	0.999973169	-2.40985263	5.3366E-05	0.01507787	-1.5686055	0.038167	0.8268165
JPH4	14	complement (23568035..23578800)	-1.743257699	0.000536228	0.091341836	-1.45114031	0.02036467	0.999973169	-2.4193236	3.9754E-08	3.2817E-05	-2.5599532	5.35E-09	5.746E-06
PAPLN	14	73237497..73274640	-1.752620295	0.007752069	0.419099906	-1.55219948	0.03699483	0.999973169	-2.60717376	5.9082E-06	0.00264183	-2.0278457	0.000824	0.096648
TMEM151B	6	44270466..44307506	-1.579415974	0.038934505	0.702818574	-1.33926266	0.18693482	0.999973169	-2.61577473	1.4768E-05	0.00546494	-2.1609027	0.000516	0.0683987

CAVIN3	11	complement (6318946..6320647)	-2.062627527	0.003688333	0.291039323	-1.41478798	0.16356639	0.999973169	-2.79781509	3.929E-05	0.01171214	-2.1005453	0.002982	0.2154967
APELA	4	164877004..164898965	-1.957188503	0.011498155	0.479193994	-1.55023913	0.09894184	0.999973169	-3.11995858	1.8768E-05	0.00664824	-1.1194256	0.671092	0.9999755
STC1	8	complement (23841915..23854807)	-3.221274543	1.61861E-06	0.001120653	-3.41303619	4.884E-07	0.00055171	-3.21506346	1.696E-06	0.00086669	-2.1323368	0.001903	0.1601316
ADRA2A	10	111077163..111080907	-4.065694981	0.000574615	0.095604382	-2.39408265	0.03134115	0.999973169	-6.02503858	1.2889E-05	0.00493994	-2.6488131	0.016613	0.564826
NPTX1	17	complement (80467148..80477843)	-3.618525942	0.000184432	0.048273962	-3.89950978	7.6205E-05	0.030860262	-7.05589272	1.3828E-08	1.4133E-05	-3.2386365	0.000635	0.0810848
RGPD1	2	86907953..87013976	-1.755610517	0.32124867	0.99997305	-2.71515125	0.07994967	0.999973169	-10.0308558	8.6554E-05	0.02265498	1.5654981	0.428104	0.9999755
NPIPA8	16	complement (18317942..18336736)	-1.174200815	0.795038898	0.99997305	1.369119656	0.61097232	0.999973169	-12.423324	8.822E-05	0.02281297	-14.532663	4.09E-05	0.0100964
CES1	16	complement (55802851..55833337)	-12.69209632	9.20771E-10	1.41161E-06	-2.20450611	0.04632508	0.999973169	-14.1662061	3.2195E-10	4.6067E-07	-1.4249462	0.370553	0.9999755
DHRS2	14	23630115..23645639	-5.962220227	0.009416832	0.440334364	-1.31140905	0.68962173	0.999973169	-14.5410688	0.00015917	0.03538441	-35.777157	3.17E-06	0.0013342
RGPD2	2	complement (87755955..87825952)	1.453874567	0.614461166	0.99997305	-1.38270838	0.66327678	0.999973169	-17.8679811	0.00018072	0.03917931	4.76523583	0.035455	0.7976523
AC008695.1	5	complement (131425891..131796983)	-1035.349249	4.23661E-05	0.016838956	-9.76802075	0.00877169	0.762444052	-176.630235	1.5872E-06	0.0008309	-1060.8512	0.000387	0.0570694
PEG3	19	complement (56810077..56840728)	-36.61107892	0.000213331	0.052628948	-898.081439	3.36E-10	1.44332E-06	-428.116793	3.1091E-09	3.9254E-06	1.12489382	0.903267	0.9999755
AL035078.4	11	31812391..32104665	-1.454269255	0.697626991	0.99997305	-1.81055499	0.53826936	0.999973169	-1250.49407	0.00011269	0.02779995	-1268.8318	0.000385	0.0570694

Supplementary Material and Methods

List of Antibodies used in the study

Antibody	Catalog Number	Manufacturer
Oct4 Rabbit	09-0023	Stemgent
Sox2 Rabbit	09-0024	Stemgent
Nanog Mouse	4903	Cell Signaling
Nanog Rabbit	4893	Cell Signaling
Klf4 Rabbit	ab215036	Abcam
Klf4 Rabbit	ab151733	Abcam
Lin 28A Rabbit	3978	Cell Signaling
Rex1 Mouse	sc-377095	Santa Cruz
TRA-1-60 Mouse	MAB4360	Millipore
TRA-1-81 Mouse	MAB4381	Millipore
GAPDH Mouse	A21994	Invitrogen
Alexa Fluor 488 Donkey Anti-Rabbit	A-21206	Thermo Fisher Scientific
Alexa Fluor 488 Donkey Anti-Mouse	A-21202	Thermo Fisher Scientific
Alexa Fluor 555 Donkey Anti-Rabbit	A-31572	Thermo Fisher Scientific
Alexa Fluor 555 Donkey Anti-Mouse	A-31570	Thermo Fisher Scientific
Donkey rabbit HRP	711-035-152	Jackson Immunological
Donkey mouse HRP	715-035-150	Jackson Immunological

Antibodies dilution used in immunocytochemistry procedure

Primary antibody	Dilution	Secondary antibody	Dilution
Oct4 (rabbit)	1:500	Alexa Fluor 488 Donkey Anti-Rabbit	1:2000
TRA1-60 (mouse)	1:200	Alexa Fluor 555 Donkey Anti-Mouse	1:2000
Sox2 (rabbit)	1:500	Alexa Fluor 488 Donkey Anti-Rabbit	1:2000
TRA-1-81 (mouse)	1:200	Alexa Fluor 555 Donkey Anti-Mouse	1:2000
Nanog (mouse)	1:500	Alexa Fluor 488 Donkey Anti-Mouse	1:2000
Lin28A (rabbit)	1:500	Alexa Fluor 555 Donkey Anti-Rabbit	1:2000
Klf4 (rabbit)	1:500	Alexa Fluor 488 Donkey Anti-Rabbit	1:2000
Rex1 (mouse)	1:200	Alexa Fluor 555 Donkey Anti-Mouse	1:2000

List of Primers

Primer name	Primer Sequence
hGAPDH F	ACGACCACTTTGTCAAGCTCATTTC
hGAPDH R	GCAGTGAGGGTCTCTCTCTTCCTCT
hSOX2 F	GGGAAATGGGAGGGGTGCAAAAGAGG
hSOX2 R	TTGCGTGAGTGTGGATGGGATTGGTG
hOCT4 F	GACAGGGGGAGGGGAGGAGCTAGG
hOCT4 R	CTTCCCTCCAACCAGTTGCCCCAAAC
hNANOG F	CATGAGTGTGGATCCAGCTTG
hNANOG R	CCTGAATAAGCAGATCCATGG
SeVF	GGATCACTAGGTGATATCGAGC
SeVR	ACCAGACAAGAGTTTAAGAGATATGTATC

RNA-Seq Library Preparation

Library preparation started with purifying and fragmentation of mRNA process. In this process, poly-T oligo attached magnetic beads purified mRNA molecules that contained polyA using two rounds of purification. Through the second elution of polyA RNA, RNA sequences were fragmented and primed for cDNA synthesis. Then, synthesis of the first strand cDNA was achieved by reverse transcription process; where cleaved RNA fragments that were primed with random hexamers were reverse transcribed into cDNA strand by reverse transcriptase and random primers. To improve strand specificity, Actinomycin D was added to the First Stand Synthesis Act D mix (FSA), which allowed RNA-dependent synthesis and prevented forged DNA-dependent synthesis. Next, the second strand cDNA was synthesized by removing the RNA template and producing a replacement strand that incorporated dUTP in place of dTTP to generate the double stranded (ds) cDNA. By the end of this process, blunt-ended cDNA was obtained which further was adenylated from its 3' ends. In the adenylation process, a single ‘A’ nucleotide was added to the 3’ ends of the blunt fragments, which would prevent them from ligating to each other during the reaction of adapter ligation. The corresponding ‘T’ nucleotide on the 3’ end of the adapter provided a complementary extension for ligating the adapter to the fragment. During the process of adapters

ligation, multiple indexing adapters were ligated to the ends of the ds cDNA in preparation for hybridization onto a flow cell.

After that, PCR amplification using PCR Primer Cocktail that annealed to the ends of the adapters was used to selectively enrich DNA fragments that have adapter molecules on both ends which would increase the amount of DNA in the library. The generated libraries were validated by performing quality control analysis and quantification of DNA library templates. Quality control analysis was done using 2100 Bioanalyzer through the use of DNA-specific chip (Agilent DNA 1000); while libraries quantification was performed by Qubit assay.
